# Diversity and distribution of the microbiome in the bulbs and rhizosphere soil of *Fritillaria thunbergii*

**DOI:** 10.3389/fmicb.2026.1752283

**Published:** 2026-03-26

**Authors:** Yang Gao, Jimeng Zhang, Liqiong Sun, Kangcai Wang, Xiaoqing Tang

**Affiliations:** College of Horticulture, Nanjing Agricultural University, Nanjing, China

**Keywords:** bulb, *Fritillaria thunbergii*, metabarcoding, microbiome, rhizosphere soil

## Abstract

As global medical resources become increasingly scarce, the demand for medicinal plants continues to rise. The growth and metabolism of medicinal plants are closely linked to rhizosphere and endophytic microorganisms. The rhizosphere soil and internal tissues of plants form stable, nutrient-rich ecosystems largely dominated by microbial communities. However, how the rhizosphere and endophytic microbiomes of *Fritillaria thunbergii* vary across geographically distinct populations, and what ecological processes shape their assembly and functional potential remain largely unexplored. We hypothesized that distinct environmental selection pressures and spatial isolation would differentially shape the assembly of bacterial and fungal communities in bulb and rhizosphere niches, and that core and unique microbial taxa play pivotal roles in shaping ecological network structure. In this study, metabarcoding was employed to investigate the bacterial and fungal communities in the rhizosphere soil and bulbs of *F. thunbergii* across four populations in China, with the aim of elucidating the biogeographic patterns, assembly mechanisms, and ecological networks of the plant-associated microbiome. The results indicate that both bacterial and fungal communities exhibited significant differences in diversity and composition across the four populations, shaped jointly by geographic isolation and environmental selection. Only a few taxa displayed both cosmopolitan distributions and high abundance, whereas most communities were distinct among ecotypes. Co-occurrence network analysis revealed that core taxa exerted stronger ecological relevance within bacterial and fungal communities compared to other ecotypes, while unique taxa played more pivotal roles in cross-domain networks. Phylogenetic analyses further uncovered microdiverse clades shaped by environmental selection, which may enhance functional resilience and contribute to the overall biogeographic patterns observed. By elucidating the biogeographic patterns and assembly mechanisms of the *F. thunbergii* microbiome, the study provides a conceptual framework for understanding plant-microbe interactions in medicinal plants and offers insights for the sustainable utilization of microbial resources in traditional medicine.

## 1 Introduction

In recent years, the demand for beneficial secondary metabolites has rapidly increased due to their high value in pharmaceutical, food, and health-related applications. These metabolites include pharmacologically active constituents from medicinal plants, microbially derived functional compounds, and plant polyphenols (Wang et al., 2025b). Medicinal plants contain a variety of bioactive molecules distributed across roots, stems, leaves, flowers, and fruits, which collectively contribute to their therapeutic properties and importance for human health (Hu et al., 2024). Microbial communities colonizing various plant niches, including bacteria, fungi, and other soil microorganisms, have emerged as key determinants of plant health, growth, and metabolic processes (Ducrocq et al., 2025; Nyimbo et al., 2025). *F. thunbergii* is a perennial herbaceous species belonging to the Liliaceae family, genus *Fritillaria*, distributed in southern Jiangsu Province, northern Zhejiang Province, and other regions of China. As a traditional Chinese medicinal herb, its primary bioactive constituents are steroidal alkaloids, including peimine and peiminine, which possess antitussive, expectorant, anti-inflammatory, and analgesic properties. Previous studies have demonstrated that microorganisms can influence the accumulation of these alkaloids in *F. thunbergii* by regulating key enzymes involved in alkaloid biosynthetic pathways (Wang et al., 2025a), suggesting that rhizosphere and endophytic microbes may play an important role in the formation of bioactive compounds in this medicinal plant. The bacterial and fungal taxa may constrain the biomass and metabolite accumulation of medicinal plants through direct effects, such as competing for carbon and other plant-derived resources, and indirect effects, including shifts in resource allocation that reflect potential growth-defense trade-offs (Jia et al., 2025; Lane et al., 2025; Wu et al., 2025). Furthermore, the taxonomic composition and functional attributes of fungal and bacterial communities often vary substantially among different plant niches, and these patterns can be shaped by geographic factors and environmental gradients (Liu et al., 2023).

Environmental variation affects the growth and metabolism of *F. thunbergii* populations (FTPs), resulting in differences in their ecosystem functions and biodiversity (Asefa et al., 2017). The rhizosphere and endophytic microbial communities are critical to the life cycle of FTPs and regulate essential ecosystem processes (Fitzpatrick et al., 2020). Despite their significance, research on the microbiomes of FTPs is limited, with existing studies largely confined to a single domain (Shi et al., 2011). However, how the rhizosphere and endophytic microbiomes of *F. thunbergii* vary across geographically distinct populations, and what ecological processes govern their assembly and functional potential, remain largely unexplored. Consequently, a comprehensive, systematic investigation of the FTP microbiome has not yet been conducted. This knowledge gap hinders the understanding of how microbial communities respond to environmentally driven differences among FTPs. To bridge this gap, it is crucial to distinguish between the rhizosphere soil and bulb endophytic microbiomes, and to clarify their compositional and structural differences.

**Figure 1:**
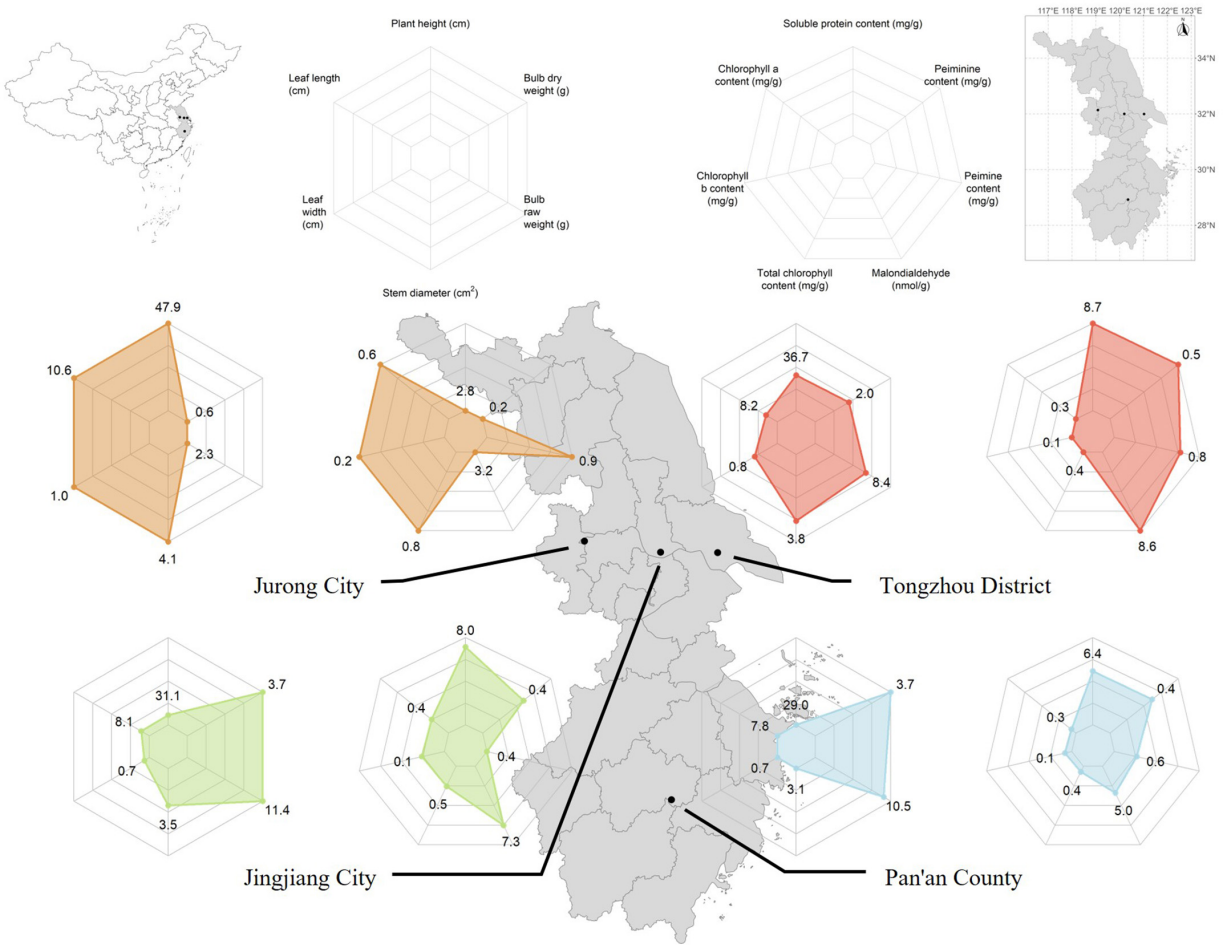
Sampling sites and plant characteristics of FTPs. Map of Jiangsu and Zhejiang Provinces showing the sampling locations (black dots) of FTPs included in this study. Radar charts illustrate the morphological traits and phytochemical profiles of FTPs, with values representing the averages of normalized parameters.

This study presents a comprehensive atlas of the bacterial and fungal microbiomes associated with the rhizosphere soil and bulbs of FTPs, and investigates the mechanisms underlying their biodiversity and biogeographical patterns. Specifically, the study aimed to characterize the diversity and composition of bacterial and fungal communities in the rhizosphere soil and bulbs of *F*. thunbergii across four geographically distinct populations, disentangle the relative roles of geographic isolation and environmental selection in shaping community assembly, and evaluate the ecological relevance of core vs. unique taxa within co-occurrence networks. To accomplish these objectives, standardized protocols were used to systematically collect rhizosphere soil and bulb endophytic microbiomes, along with botanical data, from four FTPs located in Jurong City, Jingjiang City, and Tongzhou District of Jiangsu Province, and Pan'an County of Zhejiang Province (Figure 1). In total, microbiomes were sampled from 6 rhizosphere soil samples and 6 bulb samples per population to capture the environmental heterogeneity across FTPs. Utilizing 75,218 bacterial amplicon sequence variants (ASVs) and 8,955 Kyoto Encyclopedia of Genes and Genomes (KEGG) ortholog groups (KOs), along with 9,582 fungal ASVs and 230 functional guilds classified by FUNGuild (Fungi + Functional + Guild), the taxonomic composition profiles of the microbiomes were initially compared across populations. Biogeographical and ecological analyses were then conducted based on niche- and dispersal-based community assembly theories, to disentangle the relative contributions of historical contingencies (e.g., ecological drift) and contemporary environmental selection in shaping the biodiversity and biogeography of FTP microbiomes. The findings indicate that environmental variation significantly influences both the growth of FTPs and the composition of their distinct bacterial and fungal microbiomes.

## 2 Materials and Methods

### 2.1 Study sites and sample collection

In March 2023, bulb and rhizosphere soil samples were collected from four representative FTPs in China. The sampled populations were located in Jiangsu Province (Jurong City, 32.13889 N, 119.08889 E, and Jingjiang City, 32.00111 N, 120.19389 E, and Tongzhou District, 31.99611 N, 121.0225 E), as well as Zhejiang Province (Pan'an County, 28.92445 N, 120.35167 E). At each site, six independent replicates were collected for both bulbs and rhizosphere soil to ensure adequate ecological replication. All collected plants, along with their root soil, were immediately transported back to the laboratory on dry ice. Prior to DNA extraction, all samples were stored at −80 °C.

### 2.2 Morphological characteristics and phytochemical contents

The contents of peimine and peiminine in *F. thunbergii* bulbs were precisely determined by high-performance liquid chromatography (HPLC) following the method described in the Chinese Pharmacopeia (Chinese Pharmacopoeia Commission, 2025). Fresh bulbs collected were thoroughly washed, sliced, and dried at 60 °C to a constant weight, pulverized and sieved through a No. 4 sieve (aperture = 250 μm) for HPLC analysis. Powdered samples (2 g) were moistened with 4 mL of 25% ammonia solution for 1 h, and then extracted with 40 mL of chloroform-methanol (4:1, v/v) under reflux at 80 °C for 2 h. The extract was filtered, evaporated to dryness, and redissolved in methanol for HPLC analysis using a ZORBAX Eclipse Plus C18 column (4.6 mm × 250 mm, 5 μm) with an evaporative light scattering detector (ELSD). Peimine and peiminine reference standards were used for quantification, and standard curves were established based on peak areas. Detailed chromatographic conditions are provided in the Chinese Pharmacopeia (Chinese Pharmacopoeia Commission, 2025).

### 2.3 DNA extraction, library preparation and sequencing

To collect rhizosphere soil, *F. thunbergii* roots were gently shaken by hand to remove loosely adhering soil, leaving approximately 1 mm of soil attached. The roots were then vigorously vortexed for 4 min with PBS buffer (0.1 M phosphate buffer, 0.15% Tween 80, pH 7.0). This washing step was repeated twice to ensure thorough collection of the tightly bound rhizosphere soil. The suspensions were filtered through an 80-mesh sieve to remove root tissues, and then centrifuged at 4,000 × g for 10 min. The resulting precipitated pellets were collected as the rhizosphere components. Bulbs underwent a stringent surface sterilization protocol to eliminate epiphytic microorganisms. The procedure began with a 30-s wash in sterile deionized water, followed by a 2-min immersion in 70% ethanol. Subsequently, bulbs were transferred to a 2.5% sodium hypochlorite solution supplemented with 0.1% Tween 80 for 5 min, then immersed in 70% sterile ethanol for an additional 30 s. A final series of three rinses with sterile deionized water was performed. Total DNA was extracted from the bulb and rhizosphere soil samples of *F. thunbergii*. To ensure the accuracy and reproducibility of downstream analyses, preliminary PCR optimizations were performed to determine the optimal cycle conditions. The bacterial 16S rRNA gene V5–V7 hypervariable region was amplified using primers 799F (5′-AACMGGATTAGATACCCKG-3′) and 1193R (5′-ACGTCATCCCCACCTTCC-3′), while the fungal ITS region was amplified using primers ITS1F (5′-CTTGGTCATTTAGAGGAAGTAA-3′) and ITS2R (5′-GCTGCGTTCTTCATCGATGC-3′). PCR amplifications were carried out in 20 μL reaction volumes containing 10 μL 2 × Pro Taq premix, 0.8 μL of each 5 μM forward and reverse primer, 10 ng of template DNA, and nuclease-free water to volume. Reactions were performed using an ABI GeneAmp^®^ 9700 thermal cycler with the following cycling parameters: an initial denaturation at 95 °C for 3 min; followed by 27 cycles for the 799F-1392R primer pair, or 13 cycles for the 799F-1193R primer pair, consisting of 95 °C for 30 s (denaturation), 55 °C for 30 s (annealing), and 72 °C for 45 s (extension); and a final extension at 72 °C for 10 min. Amplification success was verified by 2% agarose gel electrophoresis, with 3 μL of PCR product loaded per sample. Amplicon libraries were prepared according to Illumina MiSeq protocols, incorporating dual-index barcodes to enable high-throughput multiplexed sequencing. Sequencing was conducted by Shanghai Majorbio Bio-pharm Technology Co., Ltd. using the Illumina MiSeq platform with a paired-end 250 bp sequencing strategy.

### 2.4 Metabarcoding

In this study, a total of 96 amplicon sequence libraries were generated, consisting of 48 bulb samples and 48 rhizosphere soil samples. Paired-end sequencing produced 7,965,866 bacterial reads and 9,650,554 fungal reads, with an average of 163,335 ± 19,166 reads per sample for bacteria and 195,683 ± 44,792 reads per sample for fungi. Raw sequencing reads were initially trimmed to remove primer sequences using the *cutadapt* plugin (Martin, 2011). Subsequent sequence processing was performed using the Quantitative Insights Into Microbial Ecology pipeline, QIIME2 (version 2020.2.0; Bolyen et al., 2019). The *demux* plugin was used to visualize interactive quality profiles for read assessment. Quality filtering, denoising, dereplication, and chimera removal were performed using the DADA2 algorithm, resulting in 2,438,130 high-quality bacterial reads representing 95,196 bacterial ASVs, and 4,153,707 high-quality fungal reads representing 22,279 fungal ASVs. Taxonomic classification was performed using QIIM2's *classify-sklearn* algorithm against the SILVA reference database (v138) for bacterial ASVs and the UNITE database (v8.0) for fungal ASVs (Quast et al., 2013; Nilsson et al., 2019). Taxonomic and ASV tables, along with metadata, were subsequently imported into R (v4.5) for downstream statistical analyses. Sequences assigned to archaea, mitochondria, and chloroplasts were removed from the dataset. For each FTP, read counts were averaged across biological replicates to generate representative abundance profiles. The final ASV dataset comprised 75,218 bacterial ASVs and 9,582 fungal ASVs. Phylogenetic trees were constructed using FastTree (Price et al., 2010).

### 2.5 Data analysis

#### 2.5.1 Alpha diversity

Alpha diversity metrics, including observed ASV richness (*q* = 0) and Shannon entropy (exponentiated Shannon entropy, *q* = 1), were calculated using the *hillR* package (v0.5.2; Li, 2018). Kruskal-Wallis (KW) tests with Benjamini-Hochberg (BH) false discovery rate (FDR) correction were used to test for significant differences in microbial relative abundances among populations and niches.

#### 2.5.2 Beta diversity

Beta diversity was assessed using non-metric multidimensional scaling (NMDS) based on Bray-Curtis dissimilarity with the *vegan* package (Oksanen et al., 2025). Permutational multivariate analysis of variance (PERMANOVA) was performed using the *adonis2* function in *vegan* to test the effects of population and niche on community composition, with pairwise comparisons conducted using the *pairwiseAdonis* package (v.0.4.1; Martinez Arbizu, 2020). Multivariate generalized linear models (GLMs, *manyglm, mvabund* package, v.4.2.1) were used to validate PERMANOVA results (Wang et al., 2012; Warton et al., 2012).

#### 2.5.3 Ecotypes

**Four** ecotypes were defined at the species level. Core species were those present in both niches across all four populations with relative abundance ≥0.1% (Liu et al., 2015; Liao et al., 2017). Unique species were detected exclusively in a single niche of a single population, with mean relative abundance <0.001% for bacteria or <0.0008% for fungi (Supplementary Figure 15; Logares et al., 2013). Habitat generalists and specialists were identified based on niche breadth (Levins, 1968). The niche breadth (*B*_*j*_) for each species was calculated using Equation 1:


$$\begin{array}{l} B_{j}=\frac{1}{\sum_{i=1}^{N}P_{\left\{ij\right\}}^{2}} \end{array}$$ (1)

where *B*_*j*_ denotes the niche breadth of species *j*, and P_*ij*_ is the relative abundance of species *j* in habitat *i*. Species with observed niche breadth significantly exceeding (or falling below) null model expectations (based on 1,000 permutations, *P* < 0.05) were classified as generalists (or specialists). Enrichment of phyla within each ecotype was assessed using a binomial distribution model (Cassie, 1962; Liao et al., 2023):


$$\begin{array}{l} i=\frac{n-pN}{\sqrt{p\left(1-p\right)N}} \end{array}$$ (2)

Phyla with *i* > 2 or *i* < −2 were considered significantly overrepresented or underrepresented, respectively.

#### 2.5.4 Distance-decay patterns

Distance-decay patterns were analyzed using both taxonomic and functional datasets, based on geographic distances and various dissimilarity metrics, including Bray-Curtis, Sørensen, as well as weighted and unweighted UniFrac distances. Geographic distances between FTPs were calculated using the *distm* function from the *geosphere* package (v1.5.20), applying the *distGeo* method, which computes the shortest distance between coordinates on an ellipsoid (Hijmans et al., 2024). The statistical significance of distance-decay relationships was evaluated using Mantel tests. To compare the slopes of regression models describing distance-decay patterns, linear models were applied; when model assumptions were violated, permutational analysis of covariance was used instead.

#### 2.5.5 Co-occurrence networks

To capture deeper direct interactions, co-occurrence networks for bacteria and fungi were constructed using SpiecEasi v1.0.0 (Kurtz et al., 2015), with the neighborhood selection (MB) method, based on relative abundance data (Wen et al., 2025). Inter-domain networks were built using Spearman correlations. Network properties, including density, edge weights, node degree, betweenness, and closeness centrality, were calculated for each network. Fisher's exact tests were used to compare the proportions of positive and negative edges across populations and niches, and Kruskal-Wallis (KW) tests were used to compare centrality metrics among groups.

#### 2.5.6 Community assembly processes and phylogenetic analyses

Community assembly processes (dispersal limitation, homogeneous/heterogeneous selection, and ecological drift) were inferred using the iCAMP framework (Ning et al., 2020), which quantifies bin-level phylogenetic turnover (βNRI) and taxonomic turnover (modified Raup-Crick metric). Dominant assembly processes were visualized on phylogenetic trees using the *ggtreeExtra* package (Xu et al., 2021).

Phylogenetic signal in species contributions to beta diversity was assessed using the *adespatial and phylosignal* packages. To determine the phylogenetic depth at which beta diversity patterns emerge, ASVs were progressively agglomerated along the phylogenetic tree using tree_glom (speedyseq package) from a depth of 0–0.2 (step size of 0.005). Beta diversity (Bray-Curtis) and the number of unique phylogenetic tips were calculated at each step.

#### 2.5.7 KEGG and FUNGuild functional analyses

Bacterial KEGG profiles were predicted using the *Tax4Fun2* package (v.1.1.5; Wemheuer et al., 2020), while fungal functional guilds were assigned using the *FUNGuildR* package (v.0.3.0; Nguyen et al., 2016). The predicted KEGG counts and FUNGuild assignments for FTPs were merged in R (v.4.5.0) and log-transformed using the log1p function. To correct for differences in sequencing depth across samples, the data were normalized using total sum scaling. All subsequent statistical analyses were performed in R (v.4.5.0). Maps were generated in RStudio using the *ggplot2* package (v.3.5.2; Wickham et al., 2025).

## 3 Results

### 3.1 Morphological characteristics and phytochemical contents of *Fritillaria thunbergii* populations

Significant variation in morphological traits was observed among the FTPs. Plant height, stem diameter, and leaf area were significantly greater in the Jurong population than in the Jingjiang, Tongzhou, and Pan'an populations, whereas fresh and dry bulb weights were significantly lower (*P* < 0.05). Substantial differences in phytochemical contents were also detected across populations. Higher concentrations of chlorophyll a, chlorophyll b, total chlorophyll, and peimine were found in the Jurong population compared to the other populations, while levels of malondialdehyde, soluble protein, and peiminine were significantly lower (*P* < 0.05; Figure 1). Notably, the Jurong population represents a naturally occurring wild population, whereas the other populations are cultivated. In contrast to the cultivated populations, the Jurong population is characterized by larger aboveground organs (stems and leaves) and smaller subterranean bulbs. This population inhabits the shaded environment of Baohua Mountain in Jurong City, Jiangsu Province. The low light availability in this habitat may drive adaptive strategies that enhance photosynthetic efficiency, including increased resource allocation to aerial parts and elevated accumulation of photosynthetic pigments, thereby optimizing photosynthetic performance under resource-limited conditions.

### 3.2 FTP biodiversity patterns

**Figure 2:**
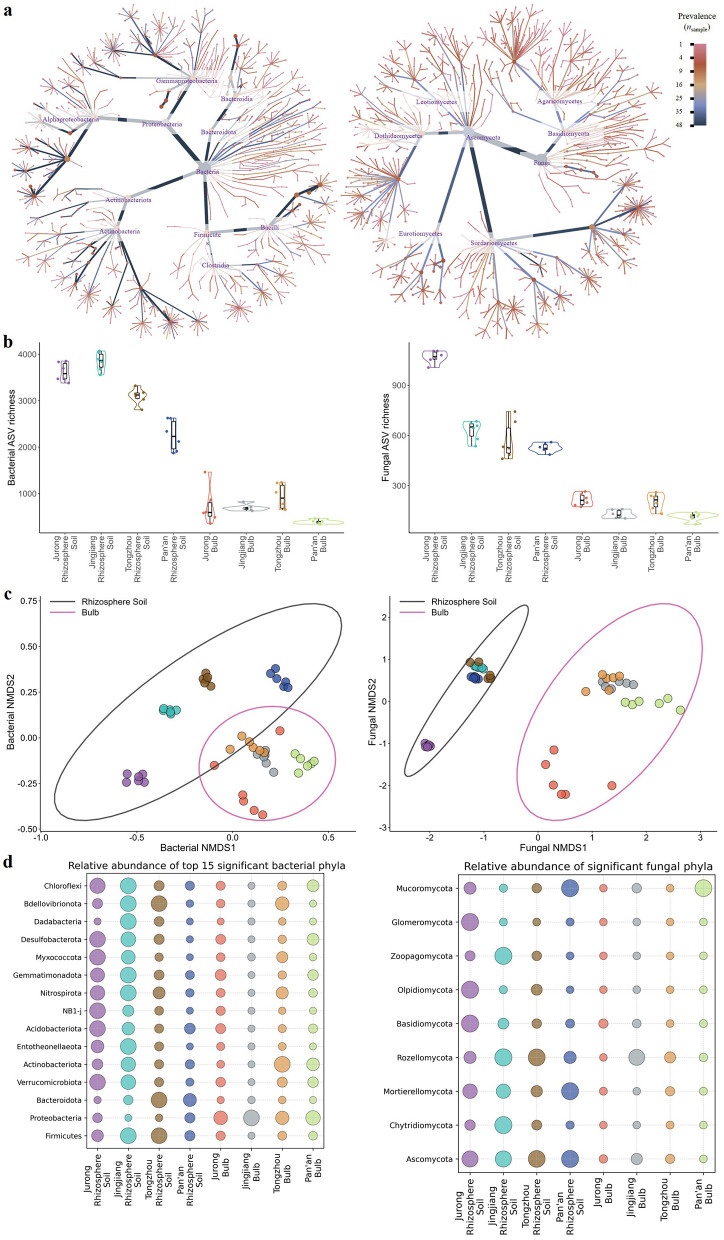
Diversity patterns of the FTP microbiome. **(a)** Heat tree depicting the taxonomic structure of bacterial and fungal communities from domain to genus level, highlighting the most prevalent lineages. Edge color indicates lineage occupancy, and node size reflects ASV frequency within each lineage. **(b)** Violin plots illustrating ASV richness across populations. Horizontal lines represent medians; box heights indicate IQR; whiskers extend to 1.5 times the IQR; each dot represents an individual sample (*n* = 48 samples). **(c)** Non-metric multidimensional scaling (NMDS) ordination based on Bray-Curtis dissimilarity, illustrating the taxonomic composition of bacterial and fungal communities across 48 samples from four populations (k = 2, stress = 0.15). Colors correspond to rhizosphere soil and bulb samples as in **(b)**. Ellipses indicate 95% confidence intervals. Bacterial community composition from rhizosphere soil, but not from bulbs, varied significantly across populations based on (1) PERMANOVA [based on 48 samples, *F*_(7, 40)_ = 3.5, *R*^2^ = 0.38, Ppopulation = 0.001) pairwiseAdonis, two-sided tests, *P*_adj_ = 0.048 or 0.028 for all tests (2) multivariate generalized linear models (*P*_adj_ <0.05 for all rhizosphere comparisons, *P*_adj_ = 0.1 for all tests from bulbs). Fungal communities differed significantly across populations [PERMANOVA: *n* = 48, *F*_(7, 40)_ = 9.1, *R*^2^ = 0.61, P_population_ = 0.001; pairwiseAdonis: *P*_adj_ = 0.048 or 0.028; manyglm: *P*_adj_ <0.05 for all tests, except for the Jingjiang Bulb and the Tongzhou Bulb]. Bacterial and fungal community composition varied significantly between rhizosphere soil and bulbs, based on bacterial PERMANOVA [*F*_(1, 46)_ = 3.7, *R*^2^ = 0.074, P_*niche*_ = 0.001], fungal PERMANOVA [*F*_(1, 46)_ = 9.6, *R*^2^ = 0.17, P_niche_ = 0.001] and both multivariate generalized linear models (two-sided tests; *P*_adj_ = 0.01). **(d)** Bubble plot showing the standardized mean relative abundances of phyla that differed significantly among FTPs (adjusted Kruskal-Wallis *P* < 0.05). To facilitate cross-ecosystem comparisons, mean relative abundances were standardized; therefore, circle sizes do not represent actual abundance values.

While the plant microbiome has received increasing attention in recent years, comprehensive insights into the bacterial and fungal biodiversity associated with FTPs remain limited. The microbial community spanned 38 bacterial and 12 fungal phyla (Figure 2a; Supplementary Figure 1a), revealing taxonomic diversity across multiple hierarchical levels. These estimates suggest that additional bacterial and fungal diversity may exist, given the conservative filtering and denoising strategies applied during data processing (see **Materials and Methods**).

Bacterial and fungal biodiversity and richness were hypothesized to be lower within the bulbs of FTPs than in the rhizosphere soil, reflecting the distinct ecological characteristics of plant tissues vs. soil habitats. Consistent with this expectation, alpha diversity analyses revealed lower diversity within bulb-associated microbiomes. The median observed bacterial richness was 1,668 ASVs (IQR: 653–3,337 ASVs; Figure 2b), with a bacterial Shannon diversity (Hill numbers) of 260.3 (IQR: 57.1–899.0 ASVs; Supplementary Figure 1b). For fungi, the median observed richness was 363 ASVs (IQR: 159–596 ASVs; Figure 2b), with a fungal Shannon diversity of 22.1 (IQR: 7.3–46.3 ASVs; Supplementary Figure 1b). These diversity values were consistently lower in bulb samples compared to rhizosphere soil, indicating that the rhizosphere provides a more heterogeneous and resource-rich environment that supports higher bacterial and fungal diversity relative to internal plant tissues (Supplementary Figures 1b, c).

Substantial variation in bacterial and fungal diversity and richness was observed among the different FTPs, suggesting that population-specific factors may further modulate microbial community assembly (Supplementary Figures 1b, c). Beta diversity analyses further revealed the spatial structure of the FTP microbiomes, revealing distinct biogeographical patterns characterized by clear separation between rhizosphere soil and bulb-associated microbiomes, as well as clustering according to population (Figure 2c).

Significant differences in relative abundance were detected across multiple taxonomic levels among the different FTPs, including 22 bacterial phyla, 45 classes, 109 orders, 174 families, 329 genera, 349 species, and 485 ASVs for bacteria, as well as 10 fungal phyla, 34 classes, 74 orders, 135 families, 233 genera, 299 species, and 294 ASVs for fungi (adjusted Kruskal-Wallis *P* < 0.05 for all taxa; Figure 2d; Supplementary Figure 2). Overall, the bacterial and fungal communities exhibited significant differences in both diversity and composition across populations and niches, likely reflecting the influence of distinct environmental conditions associated with each population and niche.

### 3.3 FTP microbiomes ecotypes

**Figure 3:**
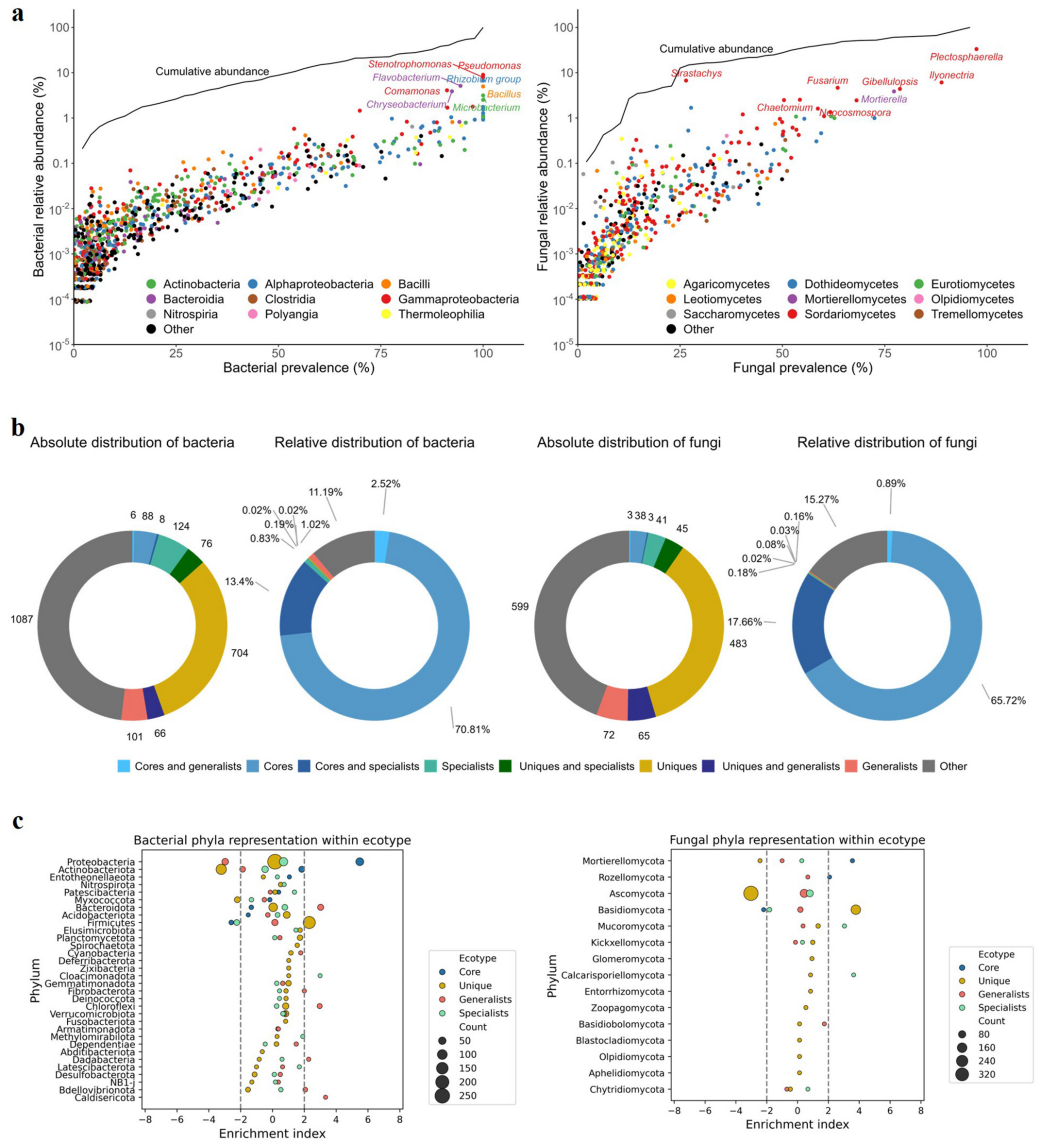
Composition and taxonomy of the FTP microbiome. **(a)** Distribution of microbial classes according to their prevalence and relative abundance. Dot color denotes class; line represents cumulative relative abundance as a function of prevalence; *y* axis represents the fraction of samples in which these taxa are present. **(b)** Absolute **(left)** and relative **(right)** abundances of the major microbiome components-specific, core, unique, and indicator ASVs-for bacterial **(left)** and fungal **(right)** communities across rhizosphere soil and bulb samples from four FTPs. **(c)** Enrichment analysis of phylum-level composition across ecotypes. Circle size represents the number of species assigned to a given phylum within each ecotype. An enrichment index > 2 or <−2 indicates significant overrepresentation or underrepresentation of the phylum, respectively, in the corresponding ecotype.

The predominant bacterial phyla included *Proteobacteria, Actinobacteriota*, and *Bacteroidota*, while *Ascomycota* dominated the fungal communities. Within these phyla, only a few bacterial genera, such as *Pseudomonas, Stenotrophomonas*, and *Bacillus*, and fungal genera, such as *Plectosphaerella, Ilyonectria*, and *Fusarium*, were highly prevalent, whereas most other genera occurred at low abundance and prevalence (Figure 3a). Bacterial and fungal species were categorized into four ecotypes: core, unique, generalists, and specialists. For both bacteria and fungi, core and unique species were differentiated by their high and low mean relative abundance across populations, respectively, while generalists and specialists were defined by their low population prevalence (Figure 3b; Supplementary Figure 3a).

For bacteria, 102 species representing 9 phyla were classified as core (Figure 3b; Supplementary Figure 3b), with Entotheonellaeota significantly enriched in this group (*P* < 0.05; Figure 3c). The unique category comprised 846 bacterial species from 31 phyla (Supplementary Figure 3c), with Firmicutes significantly enriched (*P* < 0.05; Figure 3c). A total of 173 bacterial species representing 21 phyla were identified as generalists (Supplementary Figure 3d), among which Caldisericota, Bacteroidota, and Chloroflexi were significantly enriched (*P* < 0.05 for all; Figure 3c). A similar number of bacterial species (208) were classified as specialists, but they spanned a more diverse range of 24 phyla (Supplementary Figure 3e), with significant enrichment of Cloacimonadota (*P* < 0.05; Figure 3c).

For fungi, 44 species from 4 phyla were identified as core (Figure 3b; Supplementary Figure 3b), with Mortierellomycota and Rozellomycota significantly enriched in this group (*P* < 0.05 for both; Figure 3c). The unique fungal group consisted of 593 species from 14 phyla (Supplementary Figure 3c), with Basidiomycota significantly enriched (*P* < 0.05; Figure 3c). A total of 140 fungal species representing 8 phyla were assigned as generalists (Supplementary Figure 3d), with no phyla significantly enriched within this group (*P* > 0.05). The specialist fungal group comprised 89 species from 7 phyla (Supplementary Figure 3e), with significant enrichment of Calcarisporiellomycota and Mucoromycota (*P* < 0.05 for both; Figure 3c).

The number of species in each ecotype differed significantly between the rhizosphere soil and bulb niches for both bacteria and fungi (adjusted Kruskal-Wallis *P* < 0.05 for all). The rhizosphere harbored significantly more core, unique, generalist, and specialist species compared to the bulb. Among the populations, the Jingjiang and Tongzhou populations exhibited significantly higher numbers of core, unique, generalist, and specialist bacterial species, whereas the Jurong population exhibited the highest number of unique, generalist, and specialist fungal species (Supplementary Figure 4). These results highlight the distinct ecological roles of bacterial and fungal taxa across different populations and niches.

### 3.4 Drivers of FTP microbiome composition

**Figure 4:**
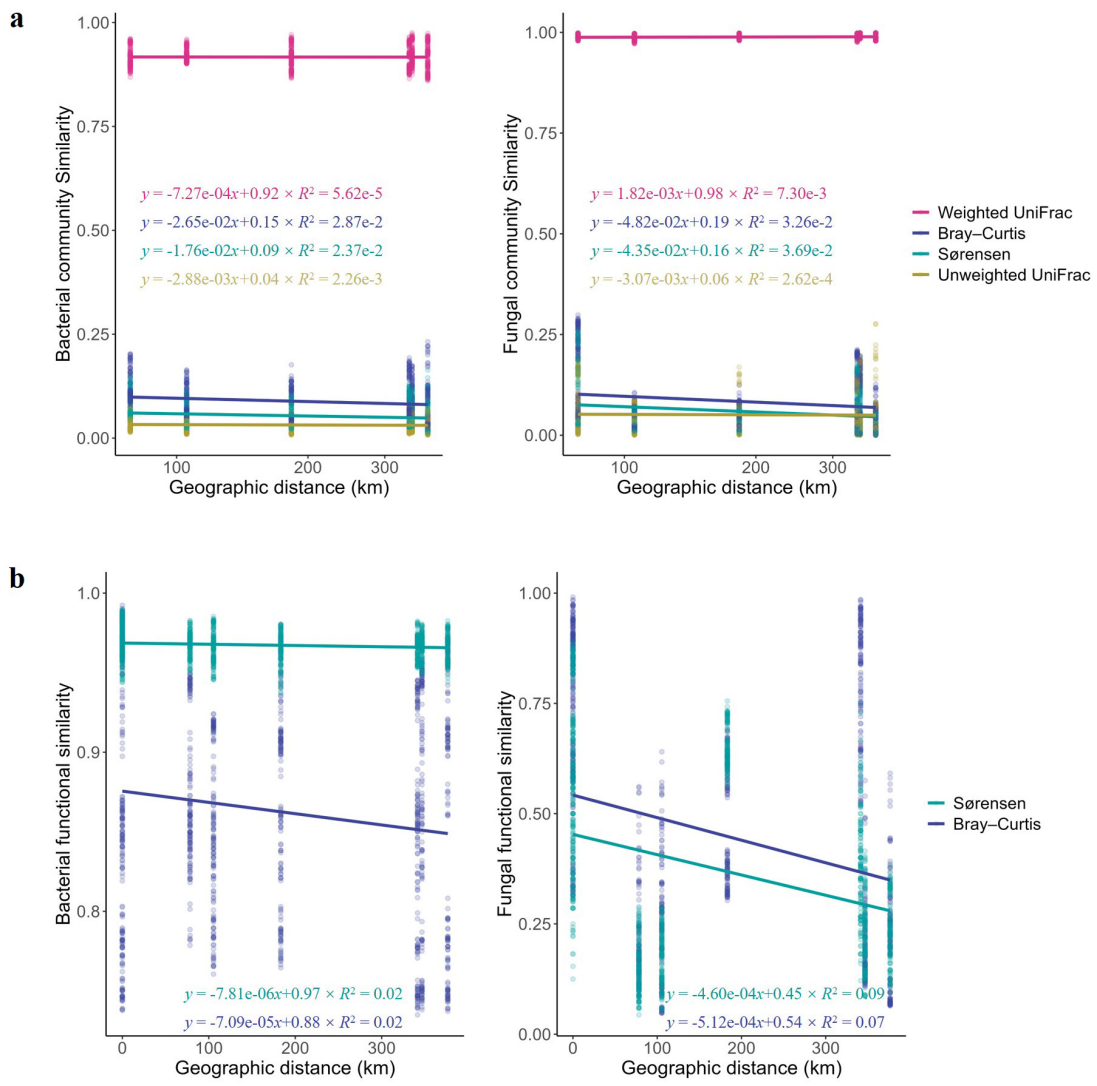
Processes driving FTP biogeographic patterns. **(a)** DDPs for the FTP bacterial and fungal microbiomes based on 48 samples and geographic distances ranging from 78 to 376 km. Similarity significantly decreased with increasing geographic distance for bacterial communities, regardless of the dissimilarity index or distance metric used (Mantel tests: *r*_BC_ = 0.46, *P*_BC_ = 0.001; *r*_SOR_ = 0.41, *P*_SOR_ = 0.001; *r*_WU_ = 0.17, *P*_WU_ = 0.01; *r*_UW_ = 0.22, *P*_UW_ = 0.001; two-sided tests; where BC is Bray-Curtis, SOR is Sørensen, WU is weighted UniFrac and UW is unweighted UniFrac).No significant differences were observed between the slopes of bacterial DDPs based on Sørensen and Bray-Curtis indices (linear model, interaction estimate = 0.00276, s.e. = 0.00359, *t* = 0.768, *P* = 0.442, adj. *R*^2^ = 0.229), nor between those based on weighted and unweighted UniFrac distances (interaction estimate = −0.00093, s.e. = 0.00120, *t* = −0.782, *P* = 0.434, adj. *R*^2^ = 0.998).In fungal communities, all metrics except weighted UniFrac showed significant distance decay with increasing geographic distance (Mantel tests: *r*_BC_ = 0.34, *P*_BC_ = 0.001; *r*_SOR_ = 0.34, *P*_SOR_ = 0.001; *r*_WU_ = 0.09, P_WU_ = 0.075; *r*_UW_ = 0.24, *P*_UW_ = 0.001; two-sided tests), indicating a significant distance-decay pattern in fungal community structure. Similarly, no significant differences were detected in fungal DDP slopes between Sørensen and Bray-Curtis indices (interaction estimate = 0.0004, s.e. = 0.0064, *t* = 0.065, *P* = 0.948, adj. *R*^2^ = 0.070), or between weighted and unweighted UniFrac distances (interaction estimate = −0.0021, s.e. = 0.0020, *t* = −1.065, *P* = 0.287, adj. *R*^2^ = 0.994). These results indicate that for both bacteria and fungi, the geographic distance-decay trend in community similarity is generally consistent across different dissimilarity metrics, lacking significant distance-by-metric interaction effects. **(b)** KOs and fungal functional groups based on 48 FTP bacterial and fungal samples. A significant decrease in similarity with increasing geographic distance was observed for bacterial functional profiles, for both Bray-Curtis and Sørensen indices (*r*_BC_KEGG_ = 0.14, *P*_BC_KEGG_ = 0.005; *r*_SOR_KEGG_ = 0.13, *P*_SOR_KEGG_ = 0.016). As a result, dissimilarity increased more rapidly with geographic distance based on Bray-Curtis compared with Sørensen (permutation tests for analysis of variance (aovperm), *F* = 10.45, *P* = 0.0016), indicating that the rate of change in dissimilarity with distance differs between the two indices. Similarly, fungal functional similarity based on FUNGuild annotations also declined significantly with increasing geographic distance (*r*_BC_FUNGuild_ = 0.26, *P*_BC_FUNGuild_ = 0.001; *r*_SOR_FUNGuild_ = 0.30, *P*_SOR_FUNGuild_ = 0.0002). aovperm analysis confirmed a significant interaction between geographic distance and dissimilarity metric (*F* = 11.94, *P* = 0.0006), again suggesting differing rates of dissimilarity increase for Bray-Curtis and Sørensen indices.

Both bacterial and fungal communities exhibit clear spatial structuring (Figure 4a). Distance-decay patterns (DDPs) based on Sørensen and Bray-Curtis dissimilarity indices indicated that ASV turnover (presence-absence) and changes in relative abundance both contribute to community differentiation over space. Phylogeny-informed analyses revealed weaker DDPs compared to taxonomic-based metrics, suggesting that closely related taxa tend to persist across geographically distant FTPs (Figure 4a). This pattern is consistent with the dominance of a limited number of taxonomic groups across populations and may reflect phylogenetically conserved ecological traits.

In contrast to the pronounced biogeographical structuring observed in the taxonomic composition of FTP bacterial and fungal communities, their functional potential, as inferred from KOs and FUNGuild assignment, exhibited much weaker spatial patterns (Figure 4b). Distance-decay analyses revealed that KO and Guild turnover remained relatively constant across geographic distances, while functional abundance exhibited only a weak spatial trend. This likely reflects the overall low dissimilarity in functional profiles across FTPs. The contrasting biogeographical patterns between community composition and functional potential suggest community-level functional redundancy, whereby different taxonomic assemblages maintain similar functional capacities. Such redundancy may be influenced by evolutionary modifications in bacterial and fungal genomes that could alter the functional repertoire of these microbial communities over time.

### 3.5 Co-occurrence networks of the FTP microbiome

**Figure 5:**
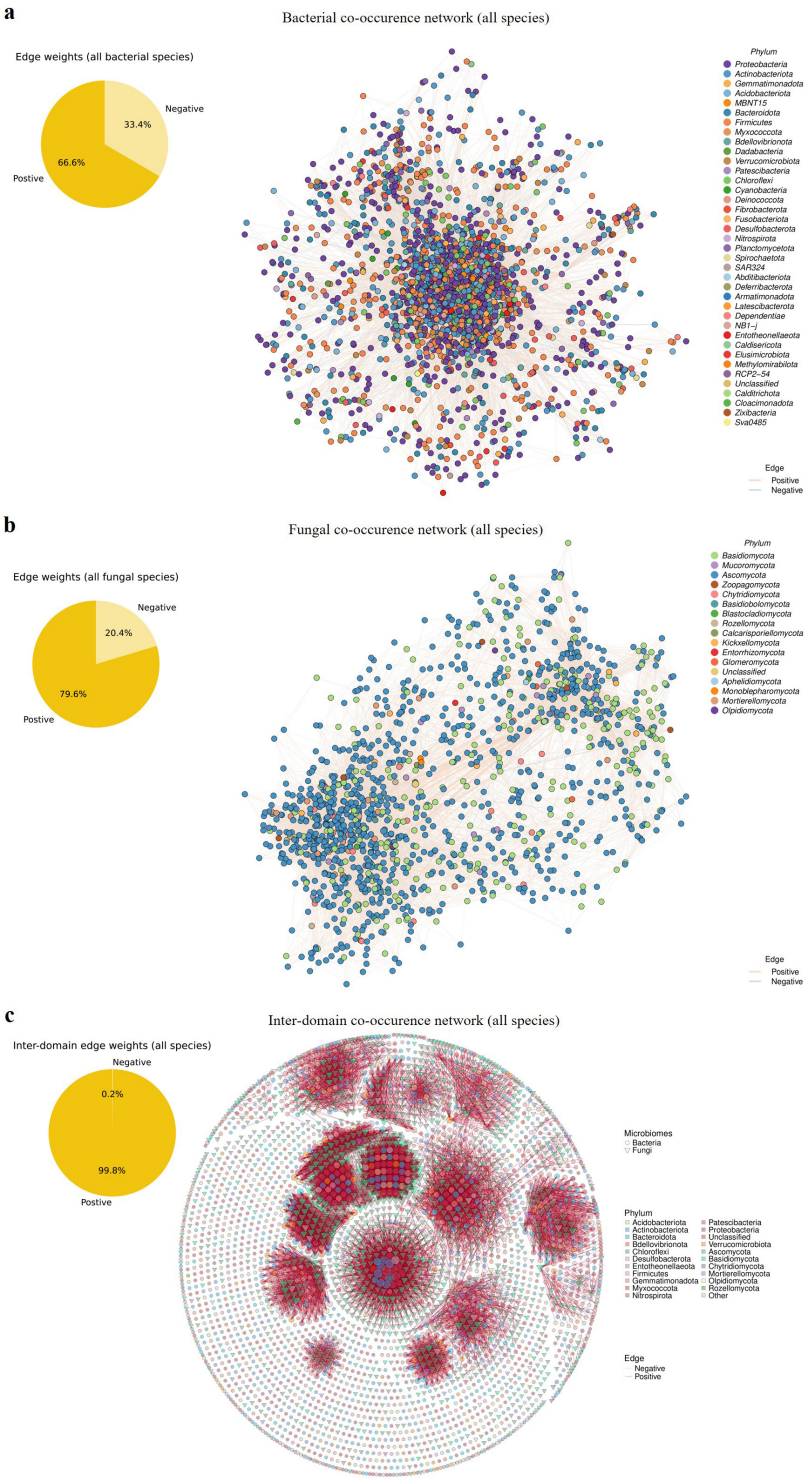
Co-occurrence network properties of bacterial and fungal communities vary across FTPs. Co-occurrence network constructed using all detected species for **(a)** bacterial communities, **(b)** fungal communities, and **(c)** inter-domain interactions. Nodes are color-coded by phylum, and edges represent significant correlations: positive edges are shown in orange, negative edges in gray.

To infer potential microbial interactions and assess the importance of taxa in shaping community connectivity and structure, co-occurrence networks were constructed for all detected species Figure 5, and node degree, betweenness centrality, and closeness centrality were calculated (Supplementary Figures 6–8).

In the bacterial network, the overall density was 0.013, with most edge weights ranging between −0.1 and 0.1 (Supplementary Figure 5). Positive edges accounted for 66.6% of total connections, substantially outnumbering negative edges (33.4%; Figure 5a). Phyla with the highest node degree included Unclassified, Abditibacteriota, MBNT15, Myxococcota, and Bdellovibrionota (Supplementary Figure 6a). The top five phyla by betweenness centrality were Sva0485, Methylomirabilota, Dadabacteria, Fusobacteriota, and Patescibacteria, while those with the highest closeness centrality were Unclassified, Calditrichota, NB1-j, Dadabacteria, and Myxococcota (Supplementary Figure 6a).

The fungal network had a density of 0.019, with most edge weights also ranging between −0.1 and 0.1 (Supplementary Figure 5). Positive correlations accounted for 79.6% of total connections, while negative correlations comprising 20.4% (Figure 5b). Phyla with the highest node degree included Unclassified, Blastocladiomycota, Basidiobolomycota, Mucoromycota, and Mortierellomycota. Mucoromycota, Kickxellomycota, Basidiomycota, Glomeromycota, and Ascomycota ranked highest in betweenness centrality, while Mortierellomycota, Monoblepharomycota, Rozellomycota, Blastocladiomycota, and Ascomycota ranked highest in closeness centrality (Supplementary Figure 7a). Taxa with both high degree and high betweenness centrality were identified as key network hubs, with Mortierellomycota and Mucoromycota designated as fungal network hubs across FTPs.

The inter-domain (bacteria-fungi) co-occurrence network had a density of 0.007, with most edge weights ranging between 0.8 and 1.0 (Supplementary Figure 5). Positive edges accounted for 99.8% of the total, indicating overwhelmingly positive interactions across domains (Figure 5c). Phyla with the highest node degree included Caldisericota, Blastocladiomycota, Cyanobacteria, Calcarisporiellomycota, and Dadabacteria. Those with highest betweenness centrality were Spirochaetota, Entorrhizomycota, Nitrospirota, Planctomycetota, and Basidiomycota, while those with highest closeness centrality were Caldisericota, Calcarisporiellomycota, Cyanobacteria, Dadabacteria, and Blastocladiomycota (Supplementary Figure 8a).

To assess the importance of different ecological types in shaping microbial community networks, co-occurrence networks stratified by ecotypes were constructed (Supplementary Figures 9a–f). In the bacterial network, core taxa exhibited significantly higher node degree centrality than unique, generalist, and specialist taxa (medians = 50.0, 12.0, 31.0, and 21.0, respectively; KW *P* = 4.76e−97; all pairwise adjusted Mann-Whitney *U* test *P* < 0.05; Supplementary Figure 6b). Generalist, specialist, and core taxa showed significantly higher betweenness centrality than unique taxa (medians = 3245.0, 2993.0, 2617.0, and 1783.9; KW *P* = 3.76e−07; all adjusted pairwise MW *P* < 0.05).Core and generalist taxa also exhibited significantly higher closeness centrality than specialist and unique taxa (medians = 8.88e−07, 7.97e−07, 6.65e−07, and 4.89e−07; KW *P* = 1.30e−105; all pairwise MW *P* < 0.05).

In the fungal network, core taxa again had significantly higher node degree than unique, generalist, and specialist taxa (medians = 39.0, 17.0, 15.0, and 13.0; KW *P* = 1.97e−16; all pairwise MW *P* < 0.05; Supplementary Figure 7b). Unique taxa showed the highest betweenness centrality compared to core, generalist, and specialist taxa (medians = 1644.0, 597.0, 1060.5, and 1463.0; KW *P* = 4.36e−02; all pairwise MW *P* < 0.05). Core taxa also exhibited significantly higher closeness centrality than all other ecotypes (medians = 1.55e−06, 1.16e−06, 1.25e−06, and 1.20e−06; KW *P* = 5.36e−22; all pairwise MW *P* < 0.05).

In the inter-domain (bacteria-fungi) network, unique taxa had significantly higher node degree than all other ecotypes (medians = 9.0, 0.0, 2.0, and 1.0; KW *P* = 8.13e−65; all pairwise MW *P* < 0.05; Supplementary Figure 8b), as well as significantly higher betweenness (medians = 1.61, 0.0, 0.125, and 0.0; KW *P* = 2.90e−19) and closeness centrality (medians = 0.56, 0.0, 0.13, and 0.13; KW *P* = 1.53e−75). These results indicate that core species play a more central ecological role in bacterial and fungal communities, while unique species are more critical in the inter-domain network.

To examine how microbial interactions vary across FTPs, separate networks were constructed for each population and niche (Supplementary Figures 10–12a–f). In the bacterial networks, the Tongzhou and Jingjiang populations' network densities were substantially lower compared to other populations and niches (Supplementary Figure 13). The proportions of positive and negative edges differed significantly across populations (Fisher's exact *P* = 0.0005), with the highest proportions of positive edges observed in the bulb (81.9%) and the lowest in the rhizosphere niche (66.2%), respectively (Supplementary Figure 6c). Significant differences were also observed in node degree, betweenness, and closeness centrality (KW *P* < 1.00e−30, <1.00e−30, and 2.02e−57, respectively). The Pan'an population exhibited the lowest node degree (median = 10.0) and betweenness centrality (median = 551.0), while the Tongzhou population had the lowest closeness centrality (median = 2.69e−06), significantly lower than all other populations and niches (all adjusted pairwise MW *P* < 0.05; Supplementary Figure 6d). In contrast, the rhizosphere niche showed the highest node degree (median = 30.0) and betweenness (median = 952.0), while the Pan'an population had the highest closeness centrality (median = 5.40e−06), significantly higher than those of all other groups except for the betweenness comparison between the Jingjiang population and the rhizosphere niche (all other MW *P* < 0.05; Supplementary Figure 6d).

In the fungal networks, the bulb niche had the lowest network density across all populations and niches (Supplementary Figure 13). Significant variation in edge polarity was also observed (Fisher's exact *P* = 0.0005), with 100.0% and 67.6% of edges being positive in the bulb and rhizosphere niches, respectively (Supplementary Figure 7c). Node degree, betweenness, and closeness centrality differed significantly among populations (KW *P* < 1.00e−30, 4.29e−58, and 3.27e−267, respectively). The bulb niche exhibited the lowest values for all three centrality metrics (medians <1.00e−30), significantly lower than those of all other populations and niches (all adjusted MW *P* < 0.05; Supplementary Figure 7d). The rhizosphere niche had the highest node degree (median = 23.0) and betweenness (median = 739.5), while the Jingjiang population showed the highest closeness centrality (median = 5.16e−06), all significantly higher than the other groups (all pairwise MW *P* < 0.05; Supplementary Figure 7d).

In the inter-domain network, the Jingjiang population had the highest overall network density (Supplementary Figure 13). The proportion of positive edges varied significantly across populations (Fisher's exact *P* = 0.0005), with the bulb niche and Pan'an population exhibiting the highest and lowest proportions (97.5% and 87.0%, respectively; Supplementary Figure 8c). Significant differences were observed in node degree, betweenness, and closeness centrality (KW *P* = 2.72e−310, 4.63e−308, and 3.31e−233, respectively). The bulb niche again exhibited the lowest values for all three metrics (medians <1.00e−30), significantly lower than all other populations and niches (all pairwise MW *P* < 0.05; Supplementary Figure 8d). In contrast, the Jingjiang population had the highest node degree (median = 15.0) and betweenness (median = 441.1), while the Jurong population had the highest closeness centrality (median = 0.31), all significantly higher than the other groups (all adjusted MW *P* < 0.05; Supplementary Figure 8d).

Collectively, these results demonstrate that the intensity and structure of microbial interactions differ across populations and niches. Bacterial communities in the Pan'an population, as well as fungal and inter-domain microbial networks in the bulb niche, exhibit markedly lower levels of connectivity compared to other populations and niches.

### 3.6 Phylogeography of the microbiome

The phylogenetic structure of ecological communities carries both evolutionary and ecological signatures that provide insights into the processes shaping biogeographical patterns. Within this framework, the phylogeography of FTP-associated bacteria and fungi was analyzed to evaluate the relative contributions of deterministic processes (i.e., environmental selection) and stochastic processes (i.e., dispersal limitation and ecological drift) in community assembly. Homogeneous environmental selection is expected to generate phylogenetic clustering, as closely related ASVs with similar ecological traits are favored under specific environmental conditions, leading to the dominance of well-adapted clades. In contrast, dispersal limitation manifests as greater compositional turnover than expected under random assembly, reflecting restricted gene flow across populations. Thus, homogeneous selection promotes community convergence and taxonomic similarity, while dispersal limitation enhances community dissimilarity and population-specificity.

**Figure 6:**
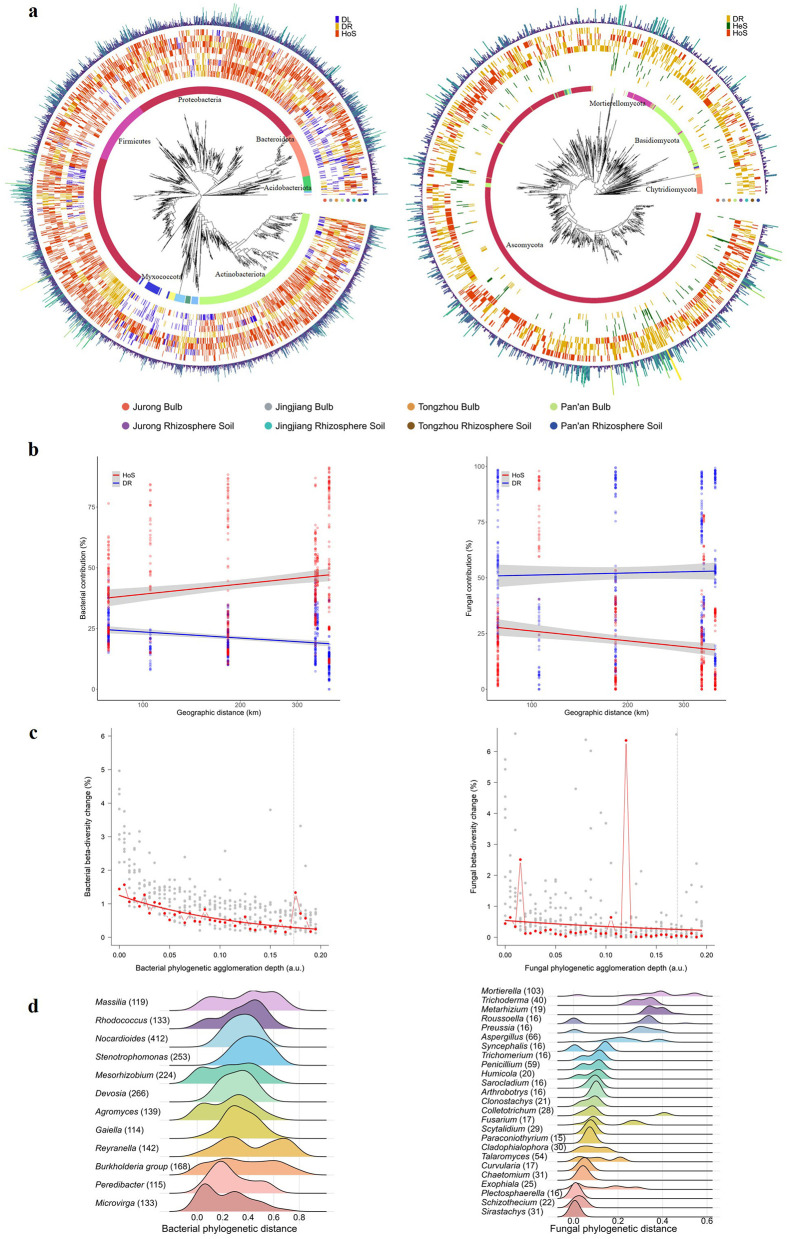
Phylogenetic structure of the FTP microbiome. **(a)** Phylogenetic tree annotated with phylum-level taxonomy. The outer rings represent the dominant bin-level community assembly processes identified for each population. In bacterial communities, homogeneous environmental selection (HoS), dispersal limitation (DL), and ecological drift (DR) were the primary processes shaping community assembly across FTPs. In contrast, fungal communities were primarily structured by HoS, DR, and heterogeneous selection (HeS). Consistent with the influence of HoS, phylogenetic clades for both bacterial and fungal taxa frequently recurred across multiple populations. **(b)** For bacteria, the contribution of HoS to community assembly increased with greater geographic distance between populations, while the influence of DR remained relatively constant across FTPs. For fungi, HoS was not strongly influenced by geographic distance, whereas the contribution of DR increased with spatial separation among populations. Solid lines and shaded areas in the figure represent the linear model fits and corresponding standard errors, respectively, for bacterial HoS (estimate_*slope*_ = −8.397, s.e. = 1.500, *t* = −5.598, *P* = 3.58 × 10^−8^, adj. *R*^2^ = 0.05688) and bacterial DR (estimate_*slope*_ = 13.857, s.e. = 3.556, *t* = 3.896, *P* = 1.11 × 10^−4^, adj. *R*^2^ = 0.02742). Similarly, the fungal HoS and DR are depicted by their respective linear fits and standard errors: for HoS (estimate_*slope*_ = 3.120, s.e. = 5.154, *t* = 0.605, *P* = 0.5452, adj. *R*^2^ = −0.001261), and for DR(estimate_*slope*_ = −14.690, s.e. = 3.776, *t* = −3.891, *P* = 1.13 × 10^−4^, adj. *R*^2^ = 0.02734). **(c)** Turnover among phylogenetically closely related ASVs was the primary contributor to both within-population (gray) and between-population (red) beta diversity. Hierarchical agglomeration of ASVs based on increasing phylogenetic distance (from terminal branches toward the root) resulted in a rapid decline in beta diversity, indicating that most compositional differences were driven by turnover among closely related taxa. The greatest reductions in beta diversity occurred during the aggregation of shallow phylogenetic branches, whereas deeper branches contributed comparatively little. For reference, the mean nearest taxon distance (MNTD) is indicated by a vertical dashed line, and fitted exponential decay models are shown as solid red lines. a.u., arbitrary units. **(d)** Clades under the influence of HoS (see **a**) include microdiverse genera. Shown are distributions of phylogenetic distances within genera that substantially contribute to relative abundance, diversity, and beta diversity within the FTP microbiome. The number of ASVs detected per genus is indicated in parentheses. Genera exhibiting tight phylogenetic clustering are considered microdiverse.

Quantification of phylogenetic and compositional turnover relative to null-model expectations (see **Materials and Methods**) revealed distinct assembly patterns for bacterial and fungal communities. In bacterial communities, homogeneous environmental selection was the predominant assembly process, governing 78.33% of community pair comparisons, followed by ecological drift (21.67%). In contrast, fungal communities exhibited a more balanced distribution of assembly processes, with homogeneous selection (46.86%) still prevailing, but with a greater contribution from ecological drift (40.59%) and a smaller role for heterogeneous selection (12.55%; Figure 6a).

For bacterial communities, homogeneous selection remained the dominant assembly process, accounting for 51.2% of the total abundance, followed by dispersal limitation (36.5%) and ecological drift (12.2%). In contrast, ecological drift was the primary assembly process in fungal communities, contributing 43.6%, followed closely by homogeneous selection (43.4%) and heterogeneous selection (10.5%; Supplementary Figure 14).

At the phylogenetic level, homogeneous selection consistently dominated several high-abundance bacterial clades, substantially contributing to total community relative abundance. Clades under homogeneous selection accounted for a median of 73.85% (IQR: 67.53%−82.36%) of relative abundance across populations, while clades shaped primarily by ecological drift contributed a median of 20.47% (IQR: 16.6%−25.84%). Genera such as *Stenotrophomonas* and *Pseudomonas* were largely shaped by homogeneous selection, whereas *Comamonas* was more strongly influenced by ecological drift. In fungal communities, ecological drift primarily structured certain clades, while homogeneous selection shaped several dominant clades that contributed substantially to overall community abundance. Clades predominantly influenced by homogeneous selection accounted for 31.76% (IQR: 29.62%−35.69%) of relative abundance per population, while clades shaped by ecological drift contributed a median of 69.73% (IQR: 66.74%−72.31%). Genera such as *Volutella* and *Sirastachys* were primarily shaped by homogeneous selection, whereas *Plectosphaerella* was predominantly structured by ecological drift.

Opposing distance-dependent trends were observed in assembly processes between bacteria and fungi. For bacteria, the influence of homogeneous selection increased with greater geographic distance between populations, while the role of ecological drift declined. Conversely, fungal homogeneous selection remained relatively constant across spatial scales, whereas the contribution of ecological drift increased with inter-population distance (Figure 6b). Together, these phylogeographic patterns further support the conclusion that both consistent environmental selection and ecological drift jointly structure the FTP microbiome.

Spatial isolation was hypothesized to promote turnover among phylogenetically closely related ASVs across FTPs. A progressive decline in bacterial beta diversity was observed with increasing phylogenetic aggregation, decreasing from a median of 0.234 (IQR: 0.212–0.261) to 0.127 (IQR: 0.122–0.131) at the deepest level of clustering. Within phylogenetic distances shorter than the mean nearest taxon distance (MNTD = 0.1732129), bacterial beta diversity declined by an average of 38.8% (IQR: 33.0–44.3%). Fungal beta diversity exhibited a similar pattern, decreasing from a median of 0.114 (IQR: 0.089–0.130) to 0.082 (IQR: 0.060–0.106), with a mean reduction of 24.1% (IQR: 21.0–27.4%) at distances below the fungal MNTD (0.1708501; Figure 6c).

The exponential decline of beta diversity in both bacteria and fungi indicates that compositional turnover predominantly occurs among closely related taxa near the tips of the phylogenetic tree. Correspondingly, the number of unique ASVs decreased exponentially with increasing phylogenetic aggregation, suggesting that many unique and niche-specific ASVs are phylogenetically clustered with taxa found in multiple populations. This pattern reflects limited dispersal over evolutionary time scales, with population-specific diversification emerging primarily among closely related microbial lineages.

Several genera subjected to homogeneous selection exhibited microdiversity, characterized by the presence of numerous ASVs separated by relatively short phylogenetic distances compared to other genera (Figure 6d). Among these microdiverse genera were key members of the FTP core bacterial microbiome, including *Stenotrophomonas* (mean intra-genus phylogenetic distance: 0.418; IQR: 0.327–0.543). Similarly, several dominant genera within the FTP core fungal microbiome also displayed pronounced microdiversity, including *Mortierella* (0.382; IQR: 0.320–0.478), *Plectosphaerella* (0.014; IQR: 0.003–0.014), and *Fusarium* (0.093; IQR: 0.088–0.255). These findings suggest that microdiverse clades may emerge through fine-scale diversification processes within lineages consistently favored by environmental selection across FTPs.

Theoretical frameworks propose that microdiversity enables the fine-scale optimization of niche space, particularly in ecosystems where environmental selection pressures are relatively weak, as is the case in FTPs. Certain clades appeared to have adapted to exploit available niche space through microdiversification, manifesting as diversification among closely related ASVs, rather than radiating into deeper phylogenetic lineages. Furthermore, the combination of fine-scale niche partitioning and dispersal limitation appeared to promote microdiversification, which ultimately contributes to the broader patterns of biodiversity and biogeography observed in FTP-associated bacterial and fungal communities.

## 4 Discussion

Overall, both bacterial and fungal communities in the rhizosphere soil and bulbs of different FTPs exhibited high diversity. Among the bacteria groups, Proteobacteria, Actinobacteriota, and Bacteroidota, together with Ascomycota in fungi, were the dominant taxa across all FTPs. These findings are consistent with recent reports on *Astragalus mongolicus, Mentha piperita*, and other medicinal plants (Shi et al., 2023; Zharkova et al., 2023). These phyla are recognized for their significant roles in global carbon cycling, primarily through the decomposition of soil organic matter and the enhancement of plant productivity, as well as for producing bioactive compounds essential to human and animal health (Spain et al., 2009; Araujo et al., 2020). Notably, Mortierellomycota and Mucoromycota-fungal groups known for their remarkable environmental adaptability and classification as extremophiles (Zhao et al., 2023; Ji et al., 2025) were identified as network hubs, indicating their crucial ecological roles within fungal communities. This finding aligns with recent studies on saline-alkali soil remediation, where Mortierellomycota was identified as a keystone taxon in fungal networks associated with salt-tolerant plants, playing critical roles in phosphorus solubilization and nutrient cycling (Cui et al., 2025). Additionally, the network analysis revealed that core taxa had a stronger ecological relevance within bacterial and fungal networks, whereas unique taxa demonstrated greater ecological centrality in cross-domain networks, as reflected by their substantially higher node degree, betweenness centrality, and closeness centrality (Supplementary Figures 6–9b). Similar micro-core microbiomes have also been reported in wastewater, freshwater, and glacial stream ecosystems (Ezzat et al., 2025; Riddley et al., 2025). Consistent with our findings, recent studies on alpine grassland restoration have shown that generalist core taxa, characterized by broad ecological niches and persistent presence, significantly contribute to network resilience by enhancing negative microbial interactions (Du et al., 2025). Although they are few in number, core taxa often exhibit high abundance, likely due to their competitive advantage. In contrast, rarity is a characteristic feature among most microbial communities, where unique taxa may act as a persistent microbial seed bank, mitigating the effects of local extinctions and dispersal while offering a broad range of ecological functions and resilience through redundancy and flexibility (Lynch and Neufeld, 2015).

In this study, homogeneous selection, a deterministic process emerged as the most prevalent and influential driver of bacterial community assembly across FTPs, resulting in phylogenetically more similar community structures among sites (Stegen et al., 2015). This finding is consistent with recent studies on *Brassica napus* and wheat rhizospheres, where homogeneous selection dominated community assembly during specific growth stages or under stress conditions (Bell et al., 2022). Consistently, we observed distance-decay relationships in bacterial communities across FTPs, a classical biogeographical pattern commonly observed in macroorganisms (Nekola and White, 1999). The interplay of deterministic and stochastic processes in microbial community assembly is widely acknowledged (Caruso et al., 2011; Dong et al., 2021); their relative contributions vary with environmental context. In this case, deterministic processes played a more prominent role in shaping fungal communities within the bulb niche. The divergence in ecological processes between bacterial and fungal communities further reflects their distinct niche adaptation strategies and dispersal capabilities.

We also found pronounced niche differentiation in the FTPs microbiome. Microbial richness and diversity were significantly lower in bulbs compared to rhizosphere soils, reflecting selective filtering imposed by distinct microenvironments (Li et al., 2025). This pattern aligns with observations in other plant systems, for instance, studies on *Casuarina equisetifolia* demonstrated that bacterial diversity and richness decreased from soils to roots to leaves (Lin et al., 2022). Microbial colonization in the rhizosphere is largely driven by rhizodeposition and relatively simple chemotactic responses to root exudates, with exudate- and mucilage-derived nutrients attracting a broad range of microorganisms (Lugtenberg and Dekkers, 1999; Walker et al., 2003; Bais et al., 2006; Lugtenberg and Kamilova, 2009; Compant et al., 2010). In contrast, successful endophytic colonization may require specific traits, such as the expression of chemotaxis-related genes, production of cell wall-degrading enzymes, and complex interactions between rhizosphere microbes and the host plant's innate immune system, which is characterized by more abrupt and dynamic fluctuations than the relatively buffered conditions in the rhizosphere (Jones and Dangl, 2006; Hardoim et al., 2008; Bulgarelli et al., 2012; Turner et al., 2013). Notably, the sources of endophytic microbiota appear to be host-dependent. In *C. equisetifolia*, fungal endophytes originate mainly from rhizosphere soil and air (Lin et al., 2022). In contrast, both bacterial and fungal endophytes in *Zingiber officinale* predominantly originate from soil, suggesting that host species identity and plant morphology strongly influence microbial recruitment pathways (Wang et al., 2024).

Collectively, this study describes the biogeographical patterns of bacterial and fungal communities in the rhizosphere soil and bulbs of four FTPs, highlighting the importance of ecological processes governing community assembly. These processes ultimately shape the heterogeneity in microbial diversity and composition across populations and niches, providing a scientific foundation for ecosystem management, improvement of medicinal plant quality, and utilization of microbial resources. Future research employing metagenomic sequencing will be essential to resolve microbial biogeography at finer scales and to enhance the understanding of plant-microbe interactions.

## Data Availability

The datasets presented in this study can be found in online repositories. The names of the repository/repositories and accession number(s) can be found at: https://www.ncbi.nlm.nih.gov/, PRJNA1359026.
