# Supplementary material for: Diversity and distribution of the microbiome in the bulbs and rhizosphere soil of Fritillaria thunbergii
Source: Front Microbiol. 2026 Mar 26;17:1752283. doi: 10.3389/fmicb.2026.1752283 (PMC13062284; doi:10.3389/fmicb.2026.1752283)
Supplement: Supplementary file 1 [file Data_Sheet_1.docx]

**
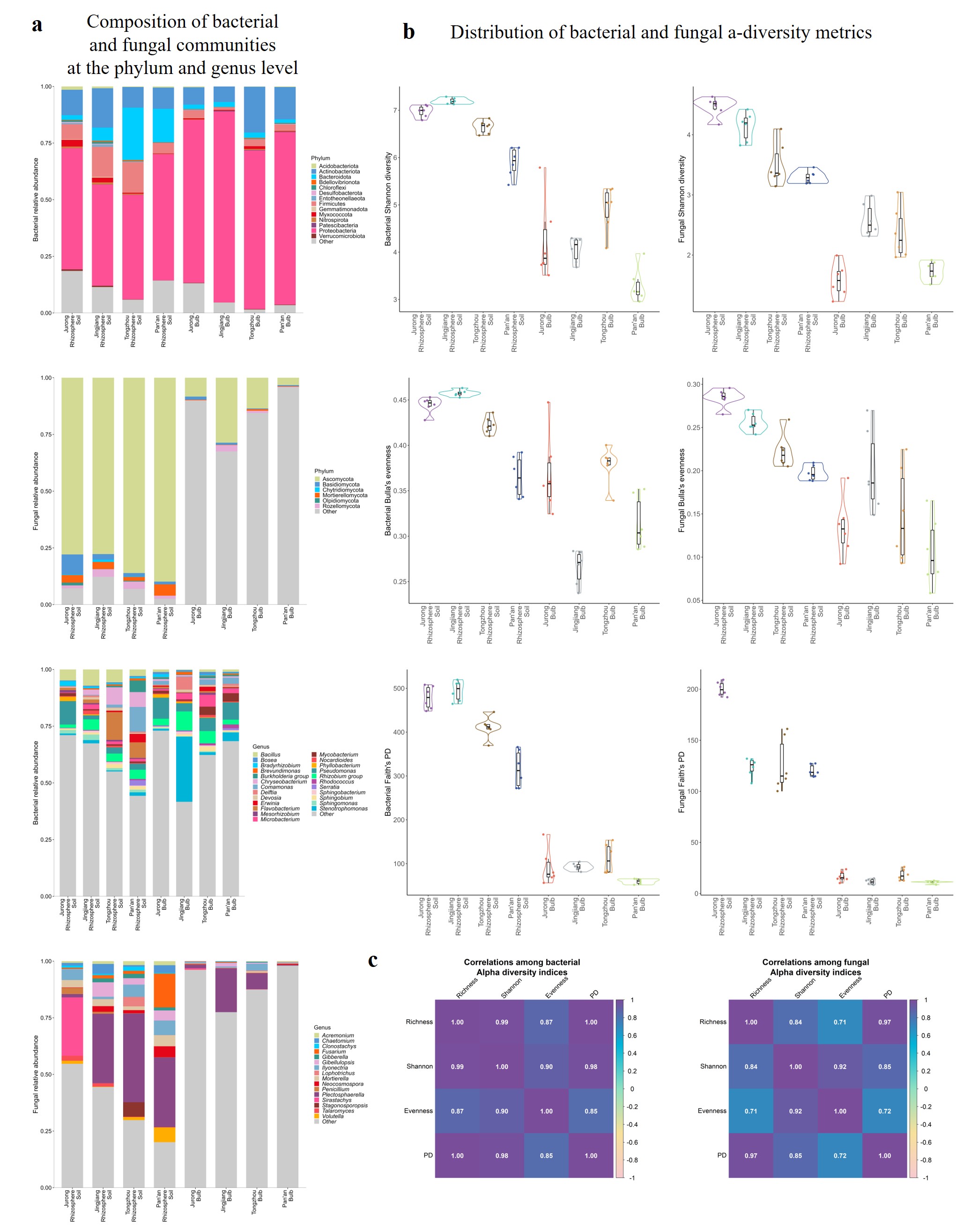
Supplementary figures**

Supplementary Fig. 1 | Composition and diversity of bacterial and fungal communities across FTPs. a, Community composition of bacteria and fungi at the phylum level for each sample. b, Histograms showing the distribution of α-diversity metrics for bacterial and fungal communities, including the Shannon-Wiener index, evenness, and Faith’s phylogenetic diversity (PD). c, Pairwise Spearman’s rank correlations among α-diversity metrics, with correlation coefficients annotated within the corresponding cells.


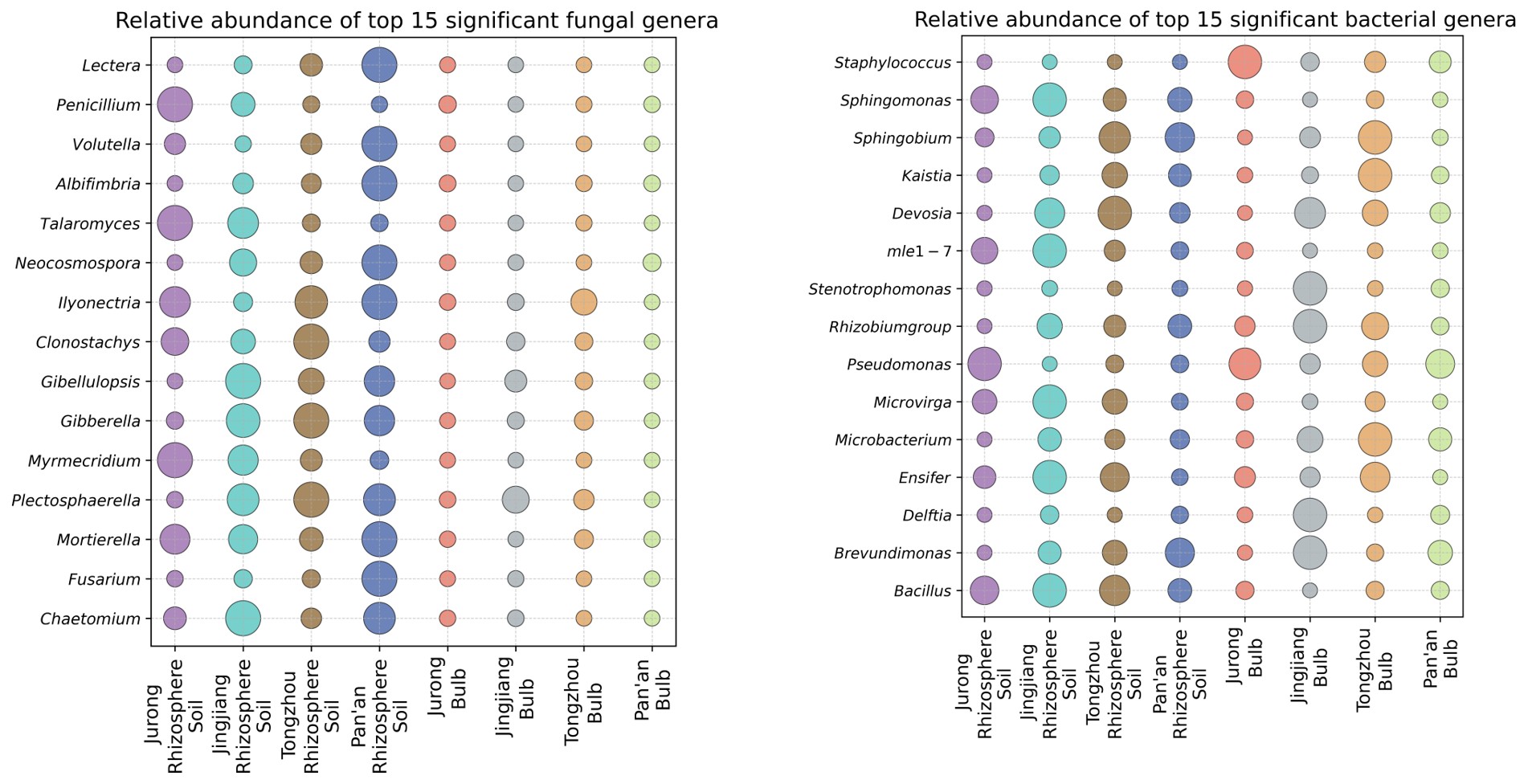
Supplementary Fig. 2 | Compositional differences of bacterial and fungal communities across FTPs. The bubble plot shows the mean relative abundance of bacterial and fungal genera that differed significantly among populations and niches (adjusted Kruskal–Wallis *P* < 0.05). Circle sizes are standardized to enhance inter-group comparison and do not reflect actual mean relative abundances.


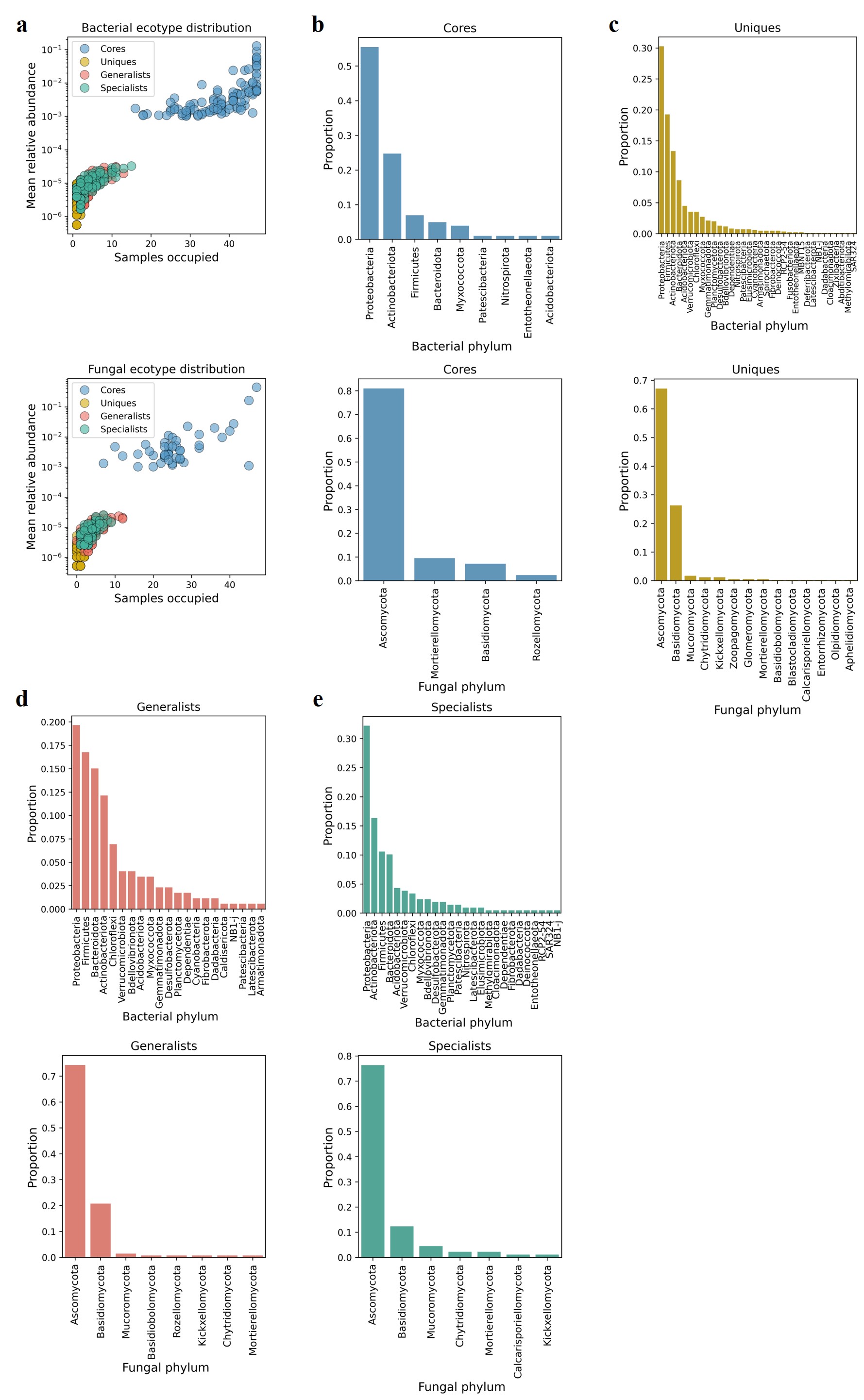
Supplementary Fig. 3 | Distribution and composition of microbial ecotypes. a, Distribution of ecotypes based on the number of samples occupied and mean relative abundance. b, c, d, e, Proportion of (b) cores, (c) uniques, (d) generalists, and (e) specialists, respectively, across different phyla, sorted in descending order.


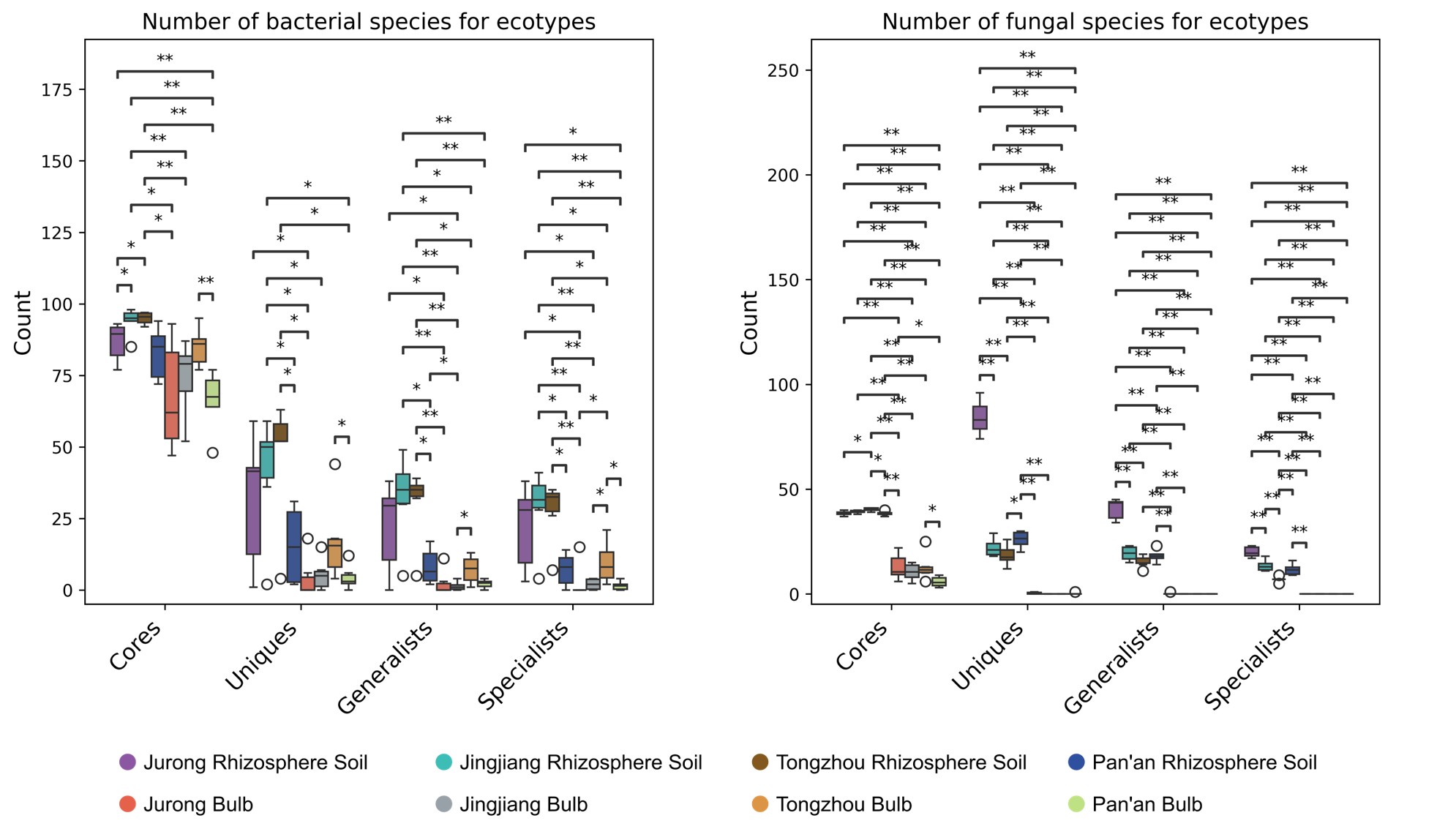
Supplementary Fig. 4 | Number of species representing each ecotype across different FTPs. In the figure, ****, ***, **, and * indicate significance levels corresponding to adjusted two-sided Mann-Whitney U test P-values < 0.0001, 0.001, 0.01, and 0.05, respectively. Only pairwise comparisons with adjusted P-values < 0.05 are displayed. The box plots show the interquartile range (IQR), with the line inside the box indicating the median and whiskers extending to 1.5 times the IQR.


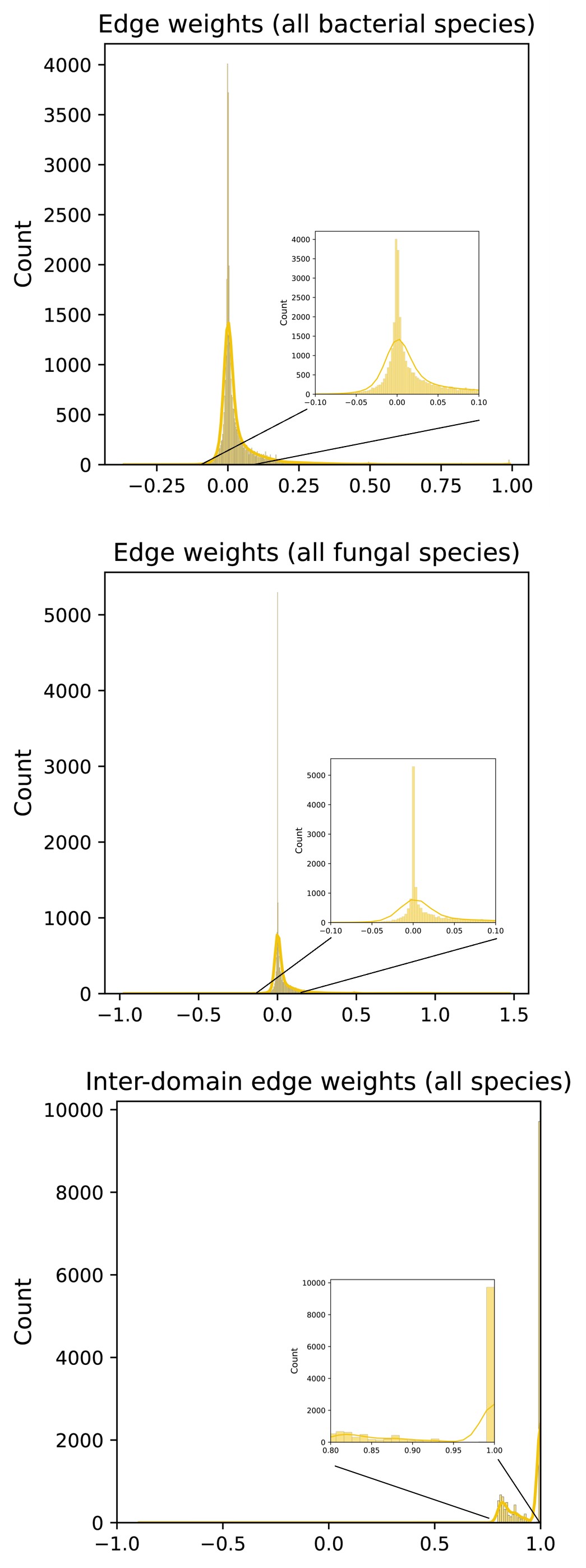
Supplementary Fig. 5 | Histogram showing the distribution of edge weights in the co-occurrence network constructed using all species. The histogram includes zoomed-in views for edge weights in the range of −0.1 to 0.1 and 0.8 to 1.0. The corresponding network is shown in Fig. 3a.


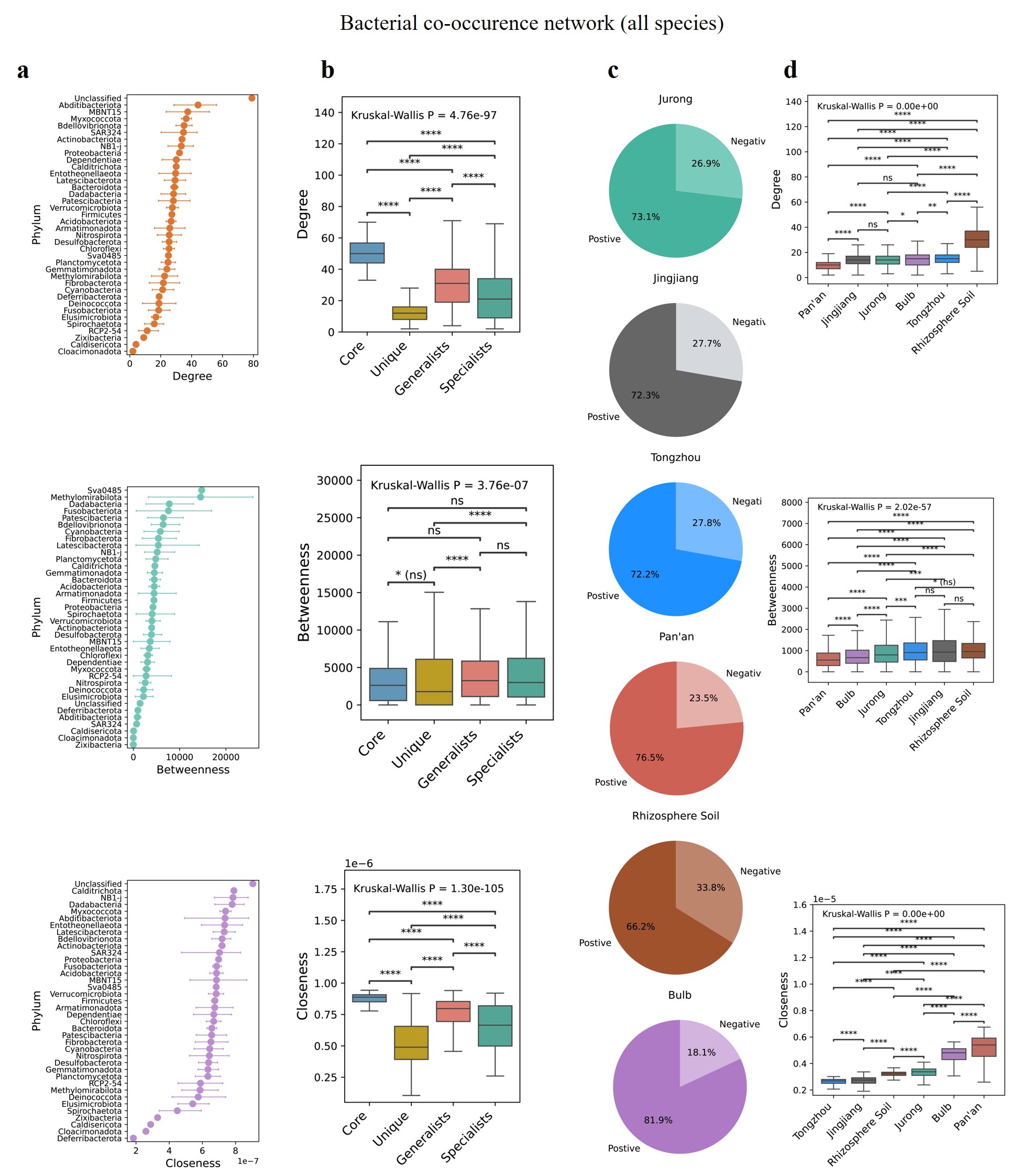
Supplementary Fig. 6 |Co-occurrence network properties of bacterial communities vary across FTPs. a. The top ten phyla ranked by node degree, closeness centrality, and betweenness centrality in the network shown in Fig. 5a, presented in descending order. Error bars represent 95% confidence intervals (mean ± 1.96 s.e.m.). b. Comparison of node degree, betweenness, and closeness centrality across ecotypes. For bacteria, *N* = 102, 846, 173, and 208 for core, unique, generalist, and specialist species, respectively. Ecotype-specific networks are shown in Supplementary Fig. 9a-b. c. Proportion of positive and negative edge weights in the co-occurrence networks of different populations and niches. The corresponding networks are shown in Supplementary Fig. 10a-f. For bacteria, *N* = 704 (Jurong), 783 (Jingjiang), 842 (Tongzhou), 435 (Pan'an), 1,298 (rhizosphere), and 630 (bulb). d. Comparison of node degree, betweenness, and closeness centrality among populations and niches, shown in ascending order. Box plots display the interquartile range (IQR), with the horizontal line indicating the median and whiskers extending to 1.5 × IQR. In (b) and (d), Kruskal-Wallis *P* < 0.05 indicates significant differences among groups. Asterisks represent adjusted two-sided Mann-Whitney *U* test *P*-values: ****, ***, **, *, and ns denote *P* < 0.0001, 0.001, 0.01, 0.05, and not significant, respectively.


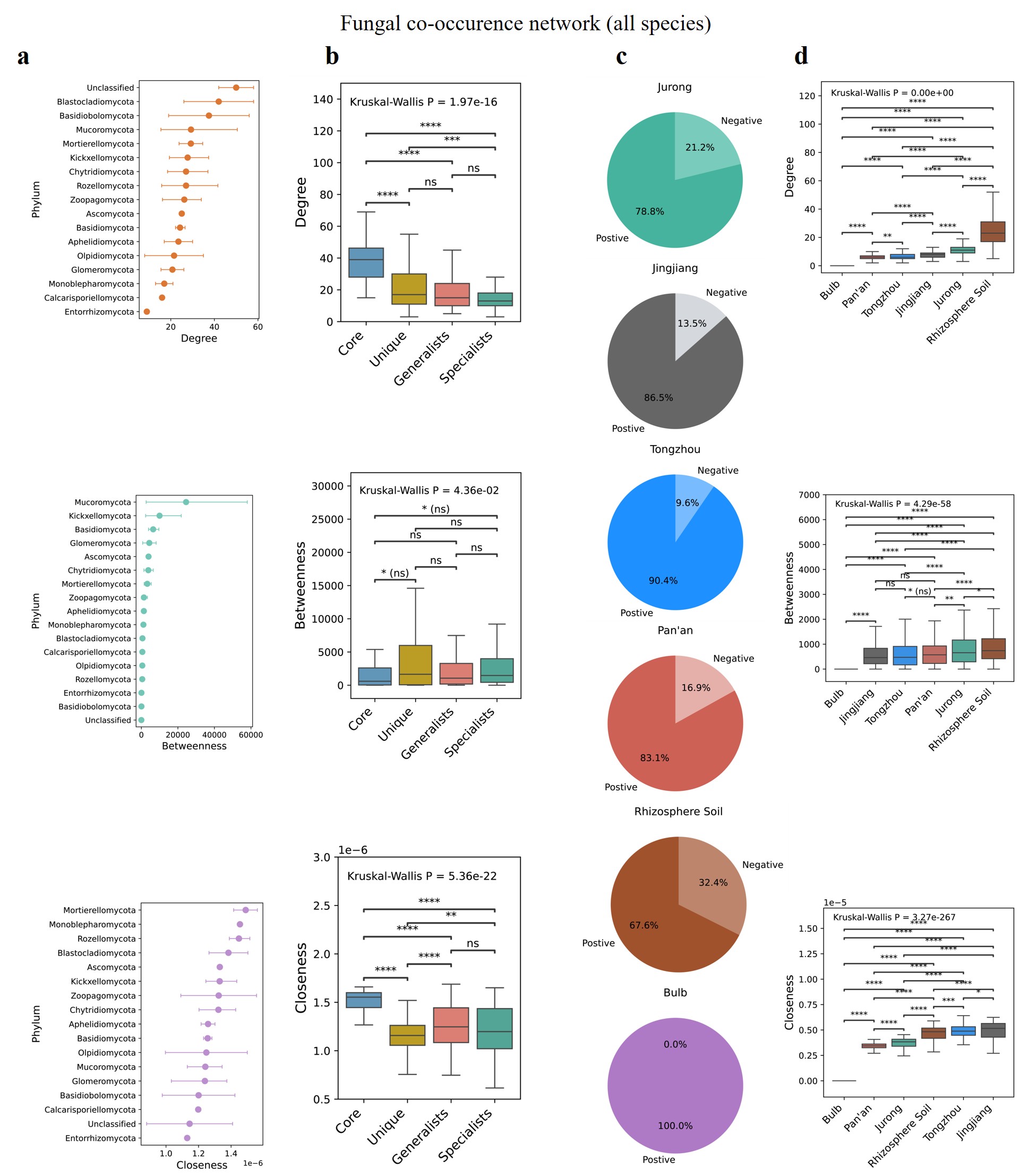
Supplementary Fig. 7 |Co-occurrence network properties of fungal communities vary across FTPs. a. The top ten phyla ranked by node degree, closeness centrality, and betweenness centrality in the network shown in Fig. 5b, presented in descending order. Error bars represent 95% confidence intervals (mean ± 1.96 s.e.m.). b. Comparison of node degree, betweenness, and closeness centrality across ecotypes. For fungi, *N* = 44, 593, 140, and 89. Ecotype-specific networks are shown in Supplementary Fig. 9c-d. c. Proportion of positive and negative edge weights in the co-occurrence networks of different populations and niches. The corresponding networks are shown in Supplementary Fig. 11a-f. For fungi, *N* = 535 (Jurong), 342 (Jingjiang), 291 (Tongzhou), 305 (Pan'an), 932 (rhizosphere), and 64 (bulb). d. Comparison of node degree, betweenness, and closeness centrality among populations and niches, shown in ascending order. Box plots display the interquartile range (IQR), with the horizontal line indicating the median and whiskers extending to 1.5 × IQR. In (b) and (d), Kruskal-Wallis *P* < 0.05 indicates significant differences among groups. Asterisks represent adjusted two-sided Mann-Whitney *U* test *P*-values: ****, ***, **, *, and ns denote *P* < 0.0001, 0.001, 0.01, 0.05, and not significant, respectively.


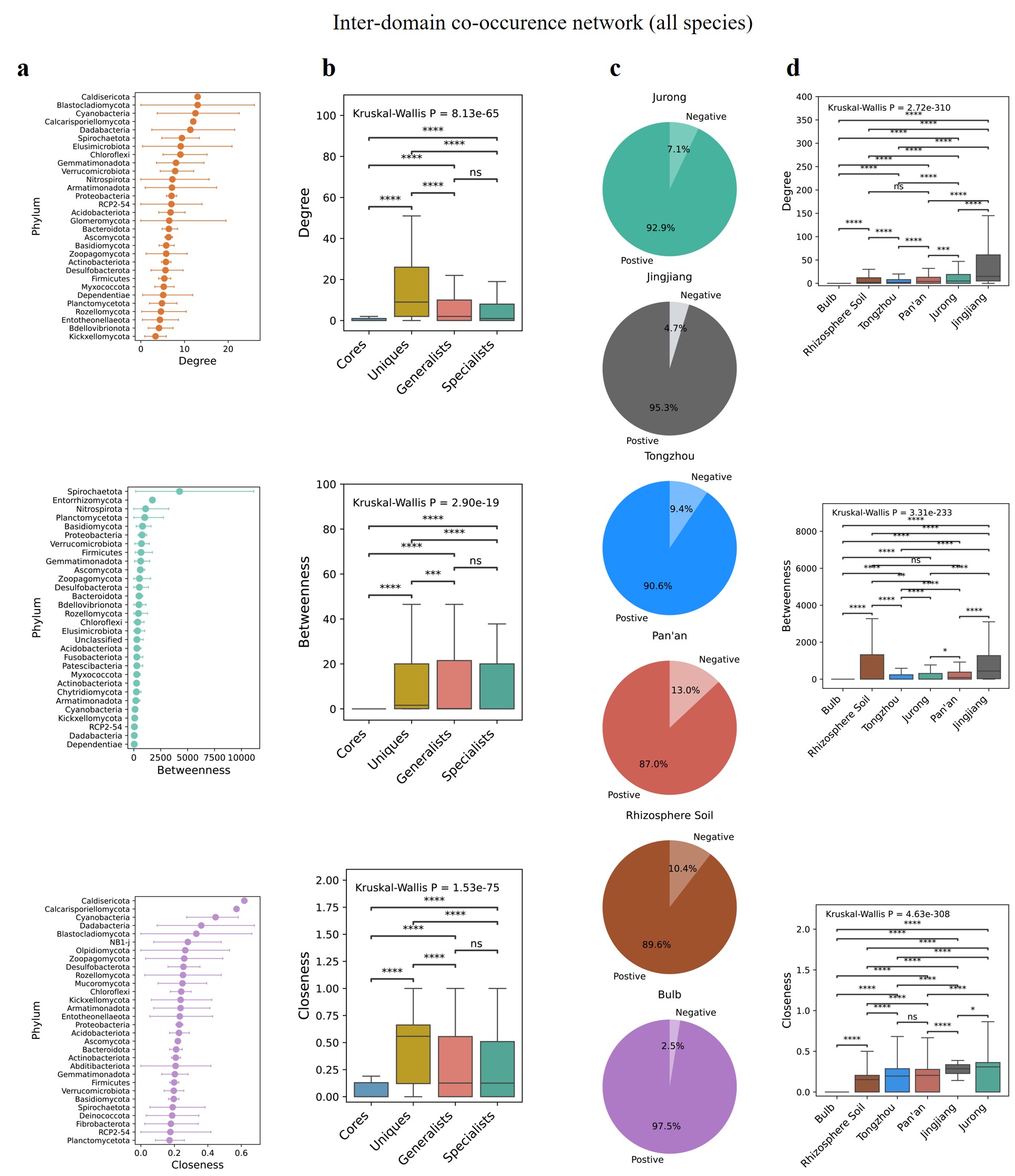
Supplementary Fig. 8 |Co-occurrence network properties of inter-domain communities vary across FTPs. a. The top ten phyla ranked by node degree, closeness centrality, and betweenness centrality in the network shown in Fig. 5c, presented in descending order. Error bars represent 95% confidence intervals (mean ± 1.96 s.e.m.). b. Comparison of node degree, betweenness, and closeness centrality across ecotypes. For inter-domain network, *N* = 146、1439、313 and 297. Ecotype-specific networks are shown in Supplementary Fig. 9e-f. c. Proportion of positive and negative edge weights in the co-occurrence networks of different populations and niches. The corresponding networks are shown in Supplementary Fig. 12a-f. For inter-domain network, *N* = 1239 (Jurong), 1125 (Jingjiang), 1133 (Tongzhou), 740 (Pan'an), 2230 (rhizosphere), and 694 (bulb). d. Comparison of node degree, betweenness, and closeness centrality among populations and niches, shown in ascending order. Box plots display the interquartile range (IQR), with the horizontal line indicating the median and whiskers extending to 1.5 × IQR. In (b) and (d), Kruskal-Wallis *P* < 0.05 indicates significant differences among groups. Asterisks represent adjusted two-sided Mann-Whitney *U* test *P*-values: ****, ***, **, *, and ns denote *P* < 0.0001, 0.001, 0.01, 0.05, and not significant, respectively.


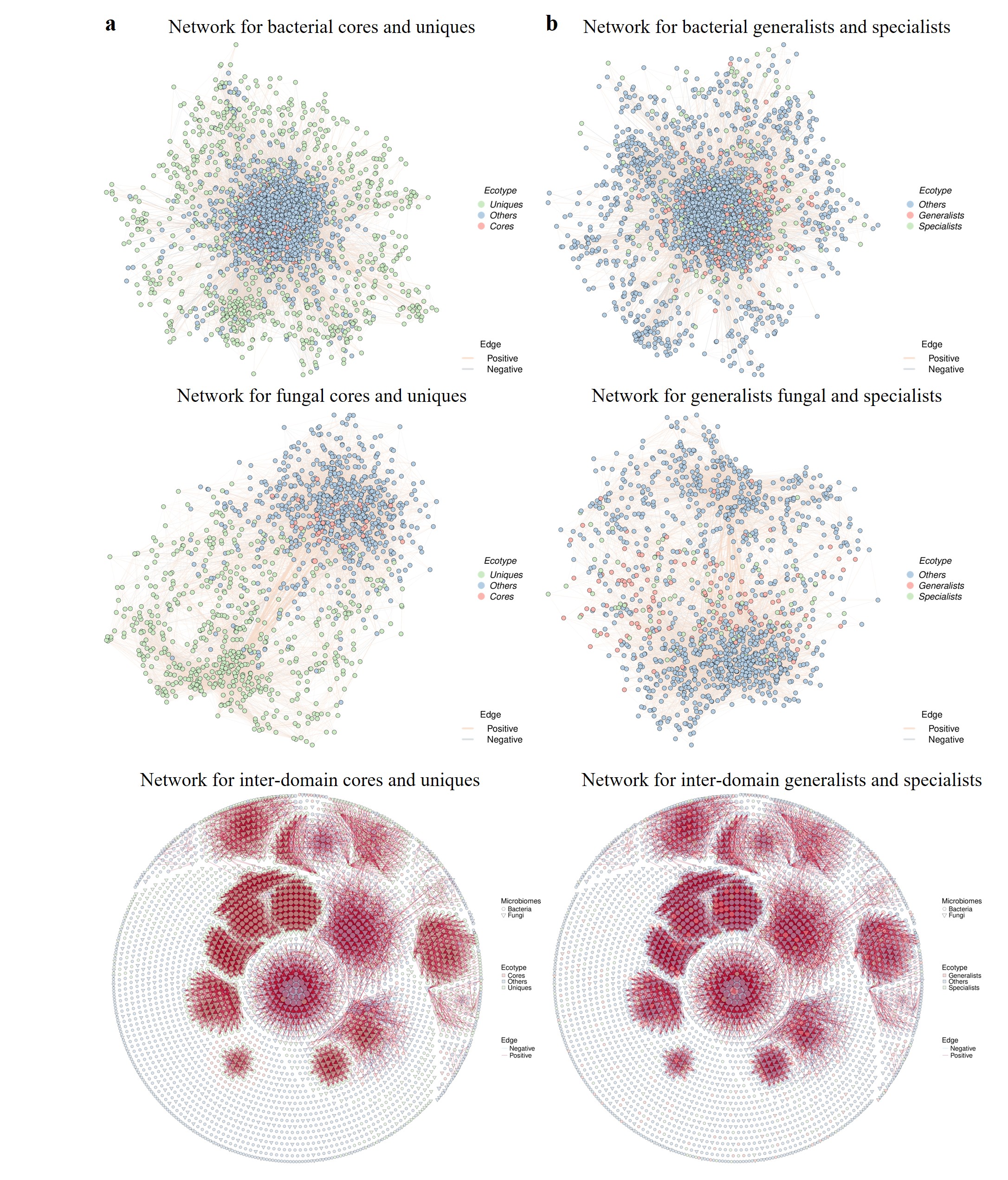
Supplementary Fig. 9| Co-occurrence networks divided by ecotypes. In (a), nodes are color-coded as core, unique, and other taxa; in (b), nodes are color-coded as generalists, specialists, and others. The networks were constructed using all species, with positive edges shown in orange and negative edges in bluish gray.


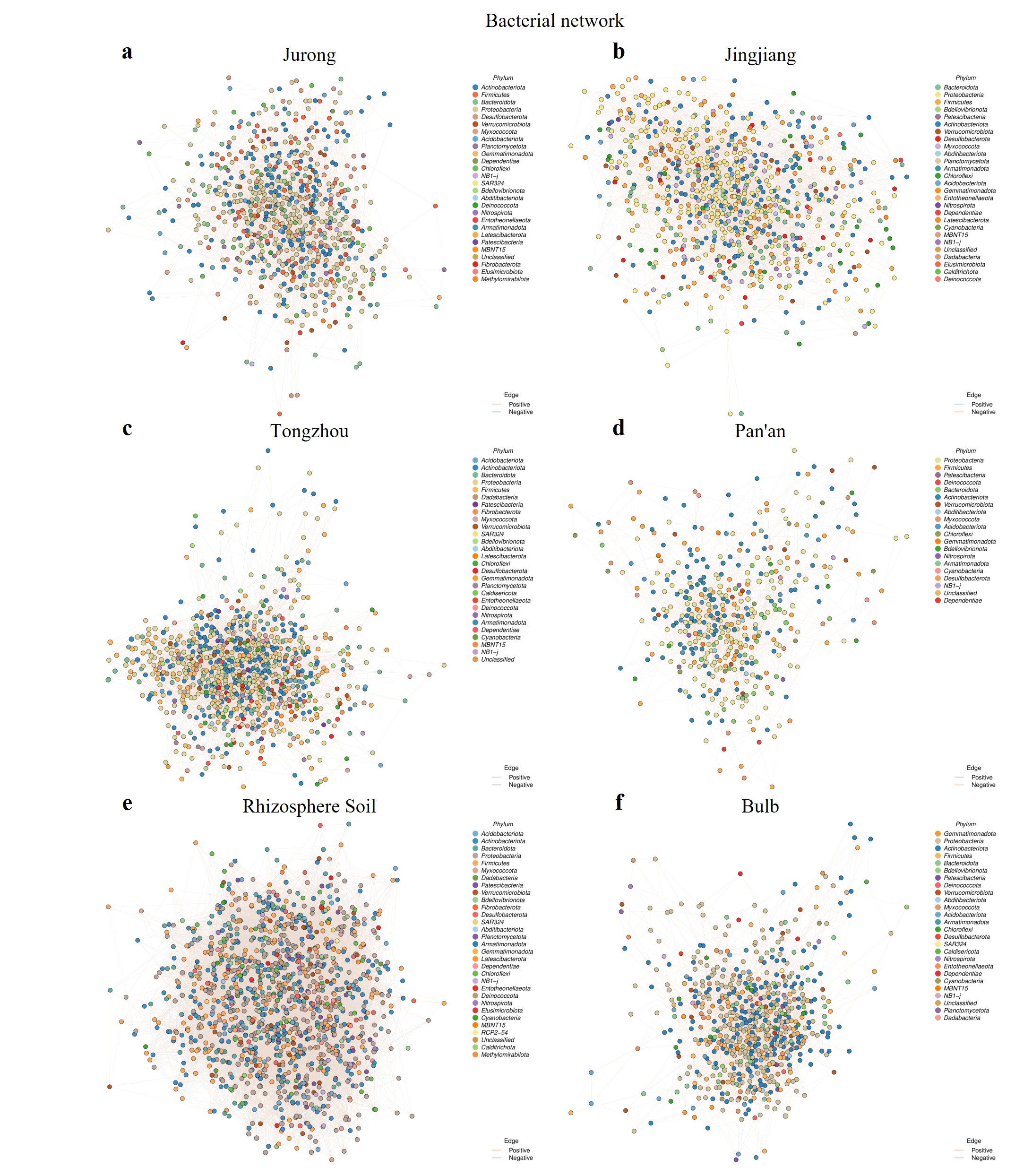
Supplementary Fig. 10 Bacterial co-occurrence networks of FTPs. Networks were constructed using species present in the following populations and niches: (a) Jurong, (b) Jingjiang, (c) Tongzhou, (d) Panan, (e) rhizosphere soil, and (f) bulb. Nodes are color-coded by phylum, with positive edges shown in orange and negative edges shown in bluish gray.


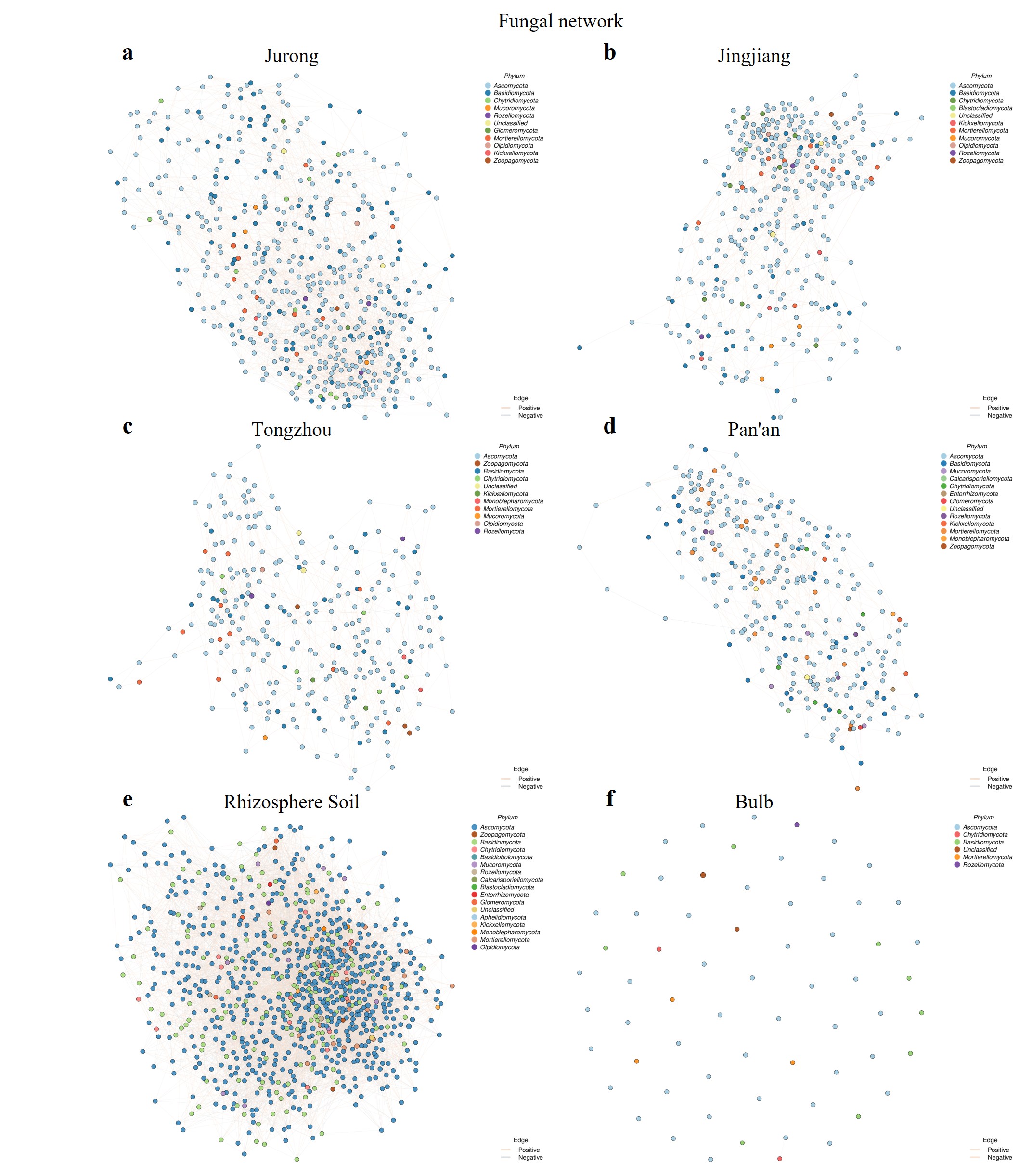
Supplementary Fig. 11 Fungal co-occurrence networks of FTPs. Networks were constructed using species present in the following populations and niches: (a) Jurong, (b) Jingjiang, (c) Tongzhou, (d) Panan, (e) rhizosphere soil, and (f) bulb. Nodes are color-coded by phylum, with positive edges shown in orange and negative edges shown in bluish gray.


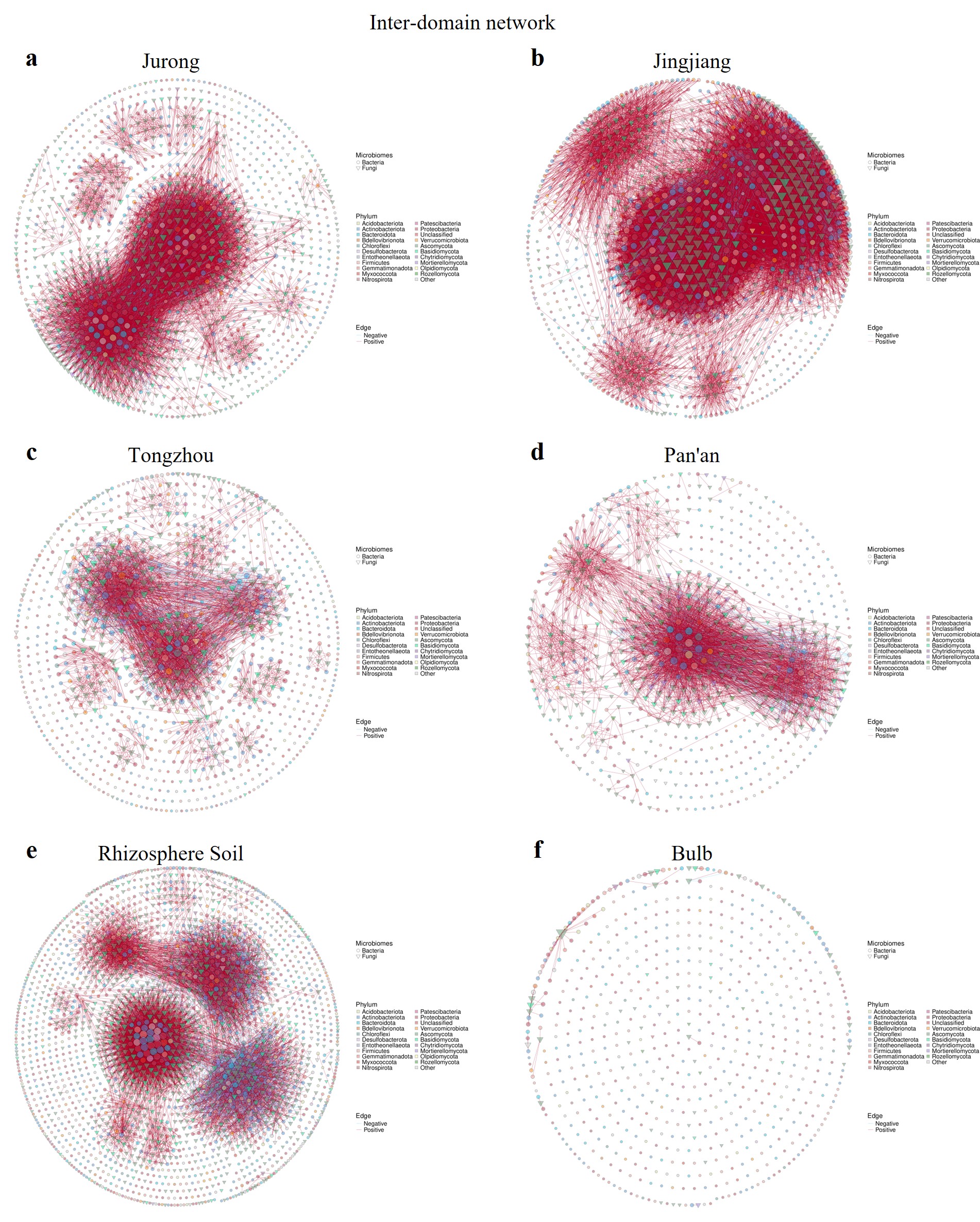
Supplementary Fig. 12 Inter-domain co-occurrence networks of FTPs. Networks were constructed using species present in the following populations and niches: (a) Jurong, (b) Jingjiang, (c) Tongzhou, (d) Panan, (e) rhizosphere soil, and (f) bulb. Nodes are color-coded by phylum, with positive edges shown in orange and negative edges shown in bluish gray.


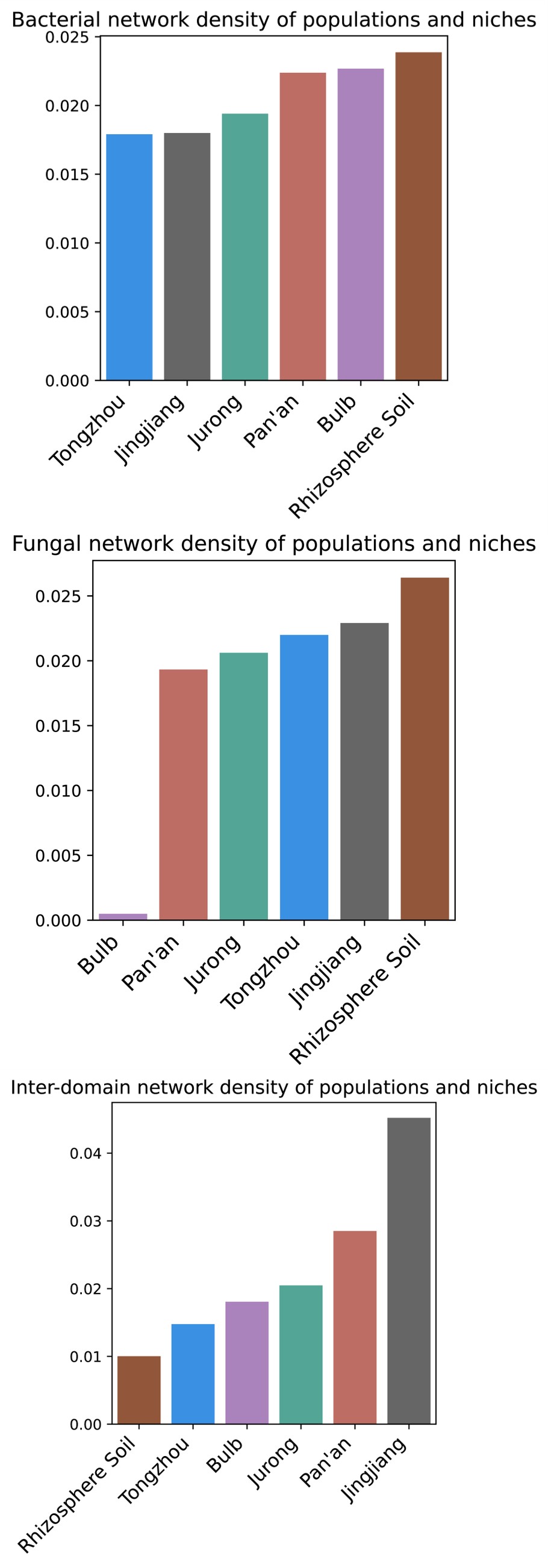
Supplementary Fig. 13| Density of co-occurrence network constructed using species present in each population and each niche. Bars are sorted in ascending order. The co-occurrence network of each population and each niche is shown in Supplementary Fig. 7-9a-f.


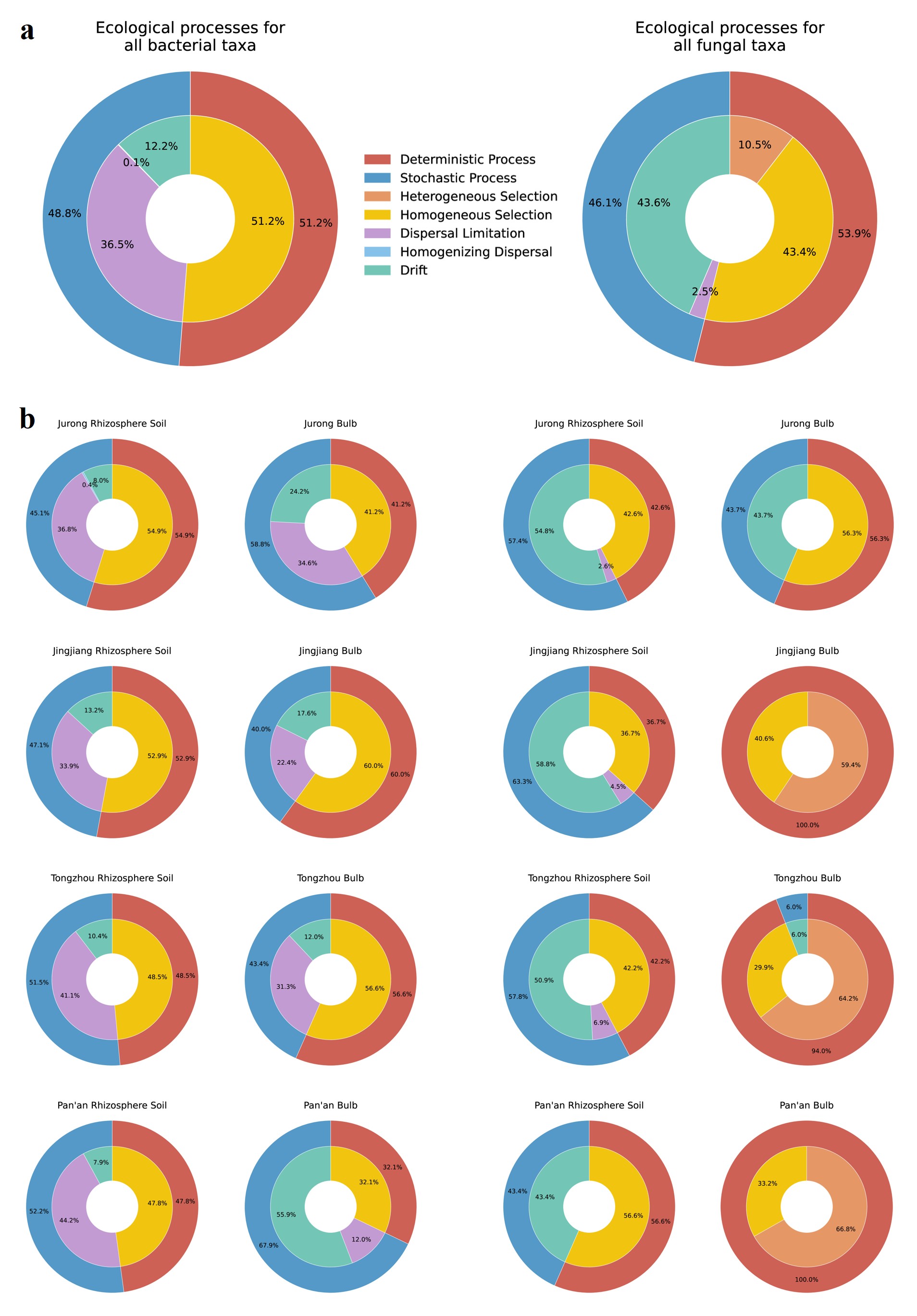
Supplementary Fig. 14| Deterministic and stochastic processes play different roles in the bacterial and fungal community assembly across FTPs. a–b Quantified the relative importance of ecological processes in shaping community structure for (a) all taxa and (b) different populations and niches. Deterministic processes include heterogeneous selection and homogeneous selection, while stochastic processes encompass dispersal limitation, homogenizing dispersal, and drift.


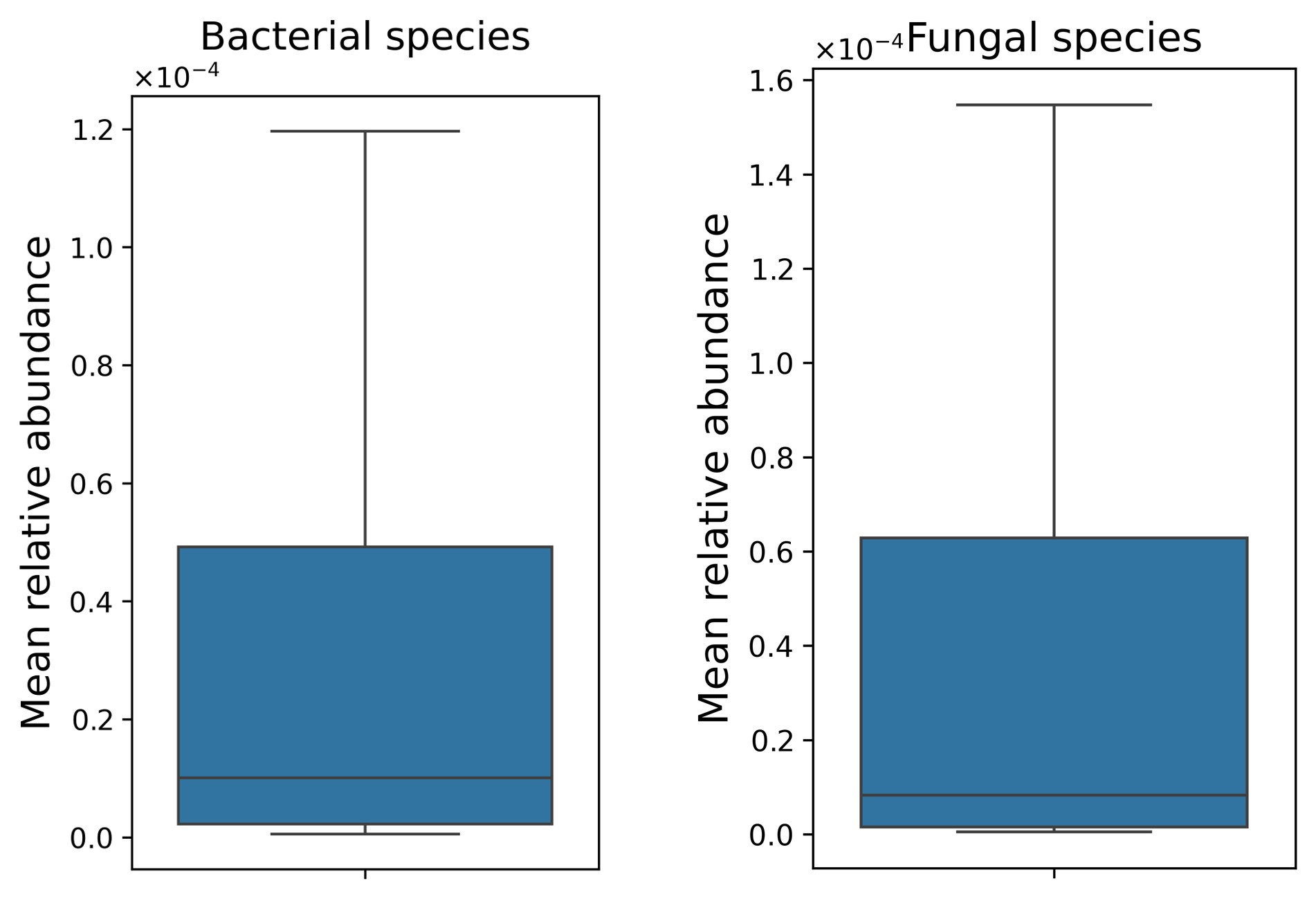
Supplementary Fig. 15| Mean relative abundance of species across samples. Box plots show the interquartile range (IQR), with the line representing the median and whiskers extending to 1.5 times the IQR.
